# Generation of functional canine TIL products for solid tumors

**DOI:** 10.3389/fimmu.2026.1810955

**Published:** 2026-05-13

**Authors:** Kay M. Foos, Veethika Pandey, Michael W. Jennings, Daniel J. Powell, Nicola J. Mason

**Affiliations:** 1 Center for Cellular Immunotherapy, Perelman School of Medicine, University of Pennsylvania, Philadelphia, PA, United States; 2 Department of Cancer Biology, Perelman School of Medicine, University of Pennsylvania, Philadelphia, PA, United States; 3 Dentistry and Oral Surgery, BluePearl Pet Hospital, Levittown, PA, United States; 4 Department of Pathology and Laboratory Medicine, Perelman School of Medicine, University of Pennsylvania, Philadelphia, PA, United States; 5 Department of Pathobiology, School of Veterinary Medicine, University of Pennsylvania, Philadelphia, PA, United States

**Keywords:** canine, cell killing, cytokines, interleukins, solid tumors, T cell activity, T cells, tumor infiltrating lymphocyte (TIL)

## Abstract

Immunotherapy based on the adoptive cell transfer of tumor-infiltrating lymphocytes (TILs) has proven effective in treating human metastatic melanoma patients, but success in tumors with lower mutational burdens remains a challenge. Preclinical evaluation of cellular therapies commonly relies on murine models, which often require implantation of tumors into immunocompromised mice and thus do not accurately reflect the complex tumor-immune interactions seen in patients. Alternatively, spontaneous tumors in client-owned dogs serve as an underutilized and valuable parallel patient population for investigating the effectiveness of adoptive cell therapy in an immunocompetent host. However, TILs have been largely unexplored in dogs. Leveraging canine cancer patients with naturally occurring low tumor mutational burden (TMB) cancer types to study TIL therapy aims to enhance preclinical translatability. To evaluate the feasibility of TIL therapy in the veterinary sector, we developed protocols to reliably expand TILs from canine oral melanoma and appendicular osteosarcoma, despite low T cell frequencies in tumor digests. A subset of these TIL products showed reactivity to autologous tumor cells from fresh tumor digests as well as early passage cell lines. Lack of TIL reactivity in a beta-2-microglobulin (B2M)-ablated canine melanoma sample confirmed that recognition was major histocompatibility complex (MHC) class I-dependent. Together, these data establish the feasibility of generating functional canine TIL products and pave the way for comparative trials to evaluate TIL efficacy and novel strategies to enhance responses in low-TMB malignancies.

## Introduction

The success of adoptive T cell therapy approaches in treating hematological malignancies has been difficult to replicate in solid tumor settings. The first FDA-approved cellular therapy for solid tumors was lifileucel, which involves adoptive cell transfer of autologous, tumor-infiltrating lymphocytes (TILs) for treatment-refractory, unresectable or metastatic melanoma (1). For this therapy, a melanoma tumor fragment is harvested and used for ex vivo expansion of TILs, which are subsequently reinfused into the patient following non-myeloablative lymphodepletion and in conjunction with high-dose interleukin-2 (IL-2) administration (2). The phase II C-144–01 trial showed an objective response rate (ORR) of 31.4%, with a subset of patients experiencing durable clinical remissions and a 5-year overall survival rate of 19.7% (2, 3). The effectiveness of TIL therapy in this context is attributable, in part, to the high tumor mutational burden (TMB) and neoantigen load of UV-driven cutaneous melanomas (4, 5). Associations between high TMB, defined as ≥10 mutations per megabase, and response to immunotherapy have been reported both within and across tumor types, as neoantigen diversity is thought to increase the likelihood of immune recognition and subsequent immunogenicity (6–9). The translation of TIL efficacy to solid tumor types with intrinsically low mutational burdens remains a challenge, as limited immunogenic neoantigen repertoire and poor immune cell infiltration can hinder TIL responses (10–12). Resistance to TIL therapy can be strengthened by the presence of an immunosuppressive tumor microenvironment (TME), often comprised of immunosuppressive cell types, immunoinhibitory molecules, insufficient costimulatory signals, and metabolic constraints (13, 14). While strategies to overcome these hurdles and to expand TIL efficacy beyond melanoma could have profound clinical implications, development has been limited by the absence of biologically relevant preclinical models that reliably recapitulate tumors with low TMB and an immunosuppressive TME.

Historically, the preclinical development of adoptive cell therapy (ACT) has depended on murine models. While these systems have provided key mechanistic insights and the development of humanized mice and patient-derived xenografts has increased their translational value, they still require implantation into immunodeficient hosts. Such a background hinders reliable demonstration of the dynamic interactions among tumor cells, the microenvironment, and the host immune system observed in human patients within a completely spontaneous, autologous setting (15). Additionally, although syngeneic mouse TIL models exist in immunocompetent mice, they are usually not spontaneous and instead depend on tumor implantation (16). Alternatively, client-owned dogs represent a valuable, yet underutilized, translational patient population that bridges this gap (17, 18). Canine tumors arise spontaneously in immunocompetent, genetically diverse hosts and share notable genetic similarities with, and are treated using therapeutics similar to, their human counterparts (18). Additionally, early proof-of-principle studies have shown that adoptive transfer approaches are feasible in dogs (19–21). Leveraging spontaneous canine cancers provides an opportunity to assess novel approaches to overcome the barriers to effective TIL therapy in a translational setting while simultaneously developing new treatment options for dogs with aggressive malignancies. However, studies that characterize canine TIL reactivity against autologous tumor cells, which serve as rationale for clinical application, are lacking.

Canine oral melanoma (OM) and appendicular osteosarcoma (OSA) are compelling tumor types for exploring TIL therapy feasibility in dogs. While human cutaneous melanoma is characterized by a predominant UV mutagenesis signature and high TMB (e.g., 14.4 mutations/Mb across 879 specimens), canine melanoma typically presents in the oral cavity, is UV-independent, and has a markedly lower TMB (~1 mutation/Mb) (22, 23). This aligns more closely with human mucosal melanoma, known for low TMB and poor response to immunotherapy (24, 25). This contrast offers a unique comparative framework to examine how TIL therapy performs in a tumor type with a shared melanocytic lineage, but under low-TMB conditions.

Canine OSA is widely recognized as a reliable translational model for human pediatric osteosarcoma, sharing clinical, biological, histological, and molecular features across species (26, 27). TMB is comparable, with approximately 1 and 1.2 mutations per megabase, for canine and humans, respectively (22, 28). Additionally, a recent large-scale transcriptomic study identified distinct TME subtypes—immune enriched, immune enriched dense extracellular matrix-like, and immune desert—that are conserved across species and are predictive of progression free survival outcomes of both canine and human patients (29). This highlights the importance of the immune cell compartment and emphasizes the translational value of canine OSA within an immunogenic context. The incidence of OSA is higher in dogs, at 27.2 per 100,000 per year, compared to 0.89 per 100,000 humans annually, making dogs both a clinically relevant and accessible population for therapeutic development (30).

Here, we demonstrate that canine OM and OSA tumors harbor T cell populations that can be ex vivo expanded using an optimized rapid expansion protocol (REP) to achieve clinically relevant numbers, and that the resulting TIL products can recognize autologous tumor cells in a major histocompatibility complex (MHC) class I-dependent manner. These findings parallel those of human TILs and thus enable pilot studies in canine patients to further the overall goal of improving therapeutic options for dogs with aggressive solid tumors and to establish a comparative, immunocompetent model to study TIL therapy in low-TMB settings with translational potential for human cancers.

## Results

### TILs can be expanded from canine oral melanoma tumor digests

**Figure 1:**
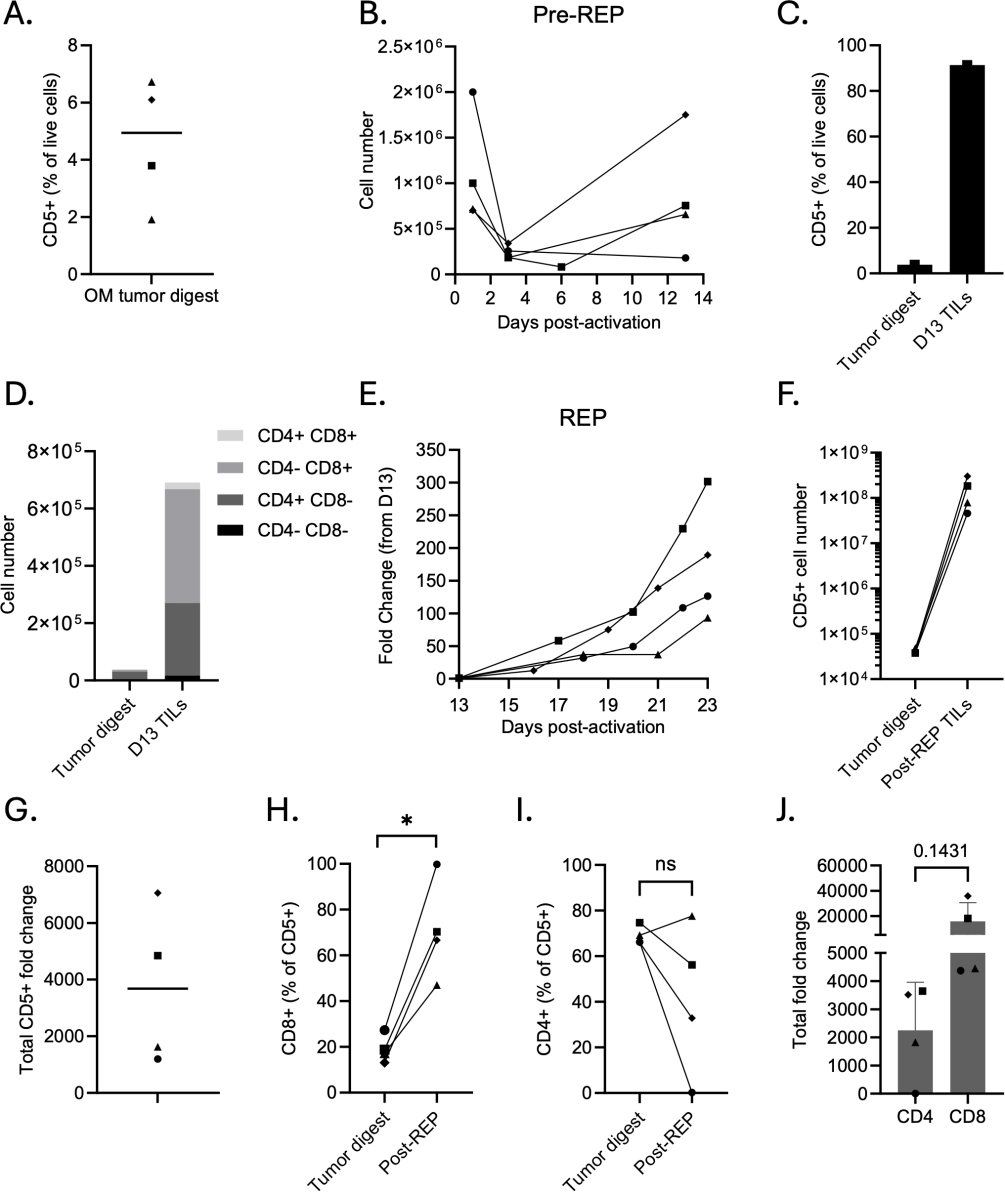
TILs can be expanded from primary canine oral melanoma tumor digests.**(A)** Quantification of flow cytometry analysis of primary tumor digests to determine the initial percentage of CD5^+^ T cells (n = 4). **(B)** Change in total cell number from oral melanoma tumor digests (day 1) during the pre-REP phase. TILs within the digests were activated with anti-dog CD3/CD28 beads in the presence of 3000 IU/mL IL-2 for 12 days. **(C)** Representative flow cytometric comparison of tumor digest and day 13 (pre-REP) TILs, demonstrating enrichment of CD5^+^ T cells during early outgrowth. **(D)** Representative analysis of CD4^+^ and CD8^+^ subsets within the CD5^+^ fraction of tumor digest and day 13 TILs **(E)** Growth kinetics of TILs during the rapid expansion protocol (REP). TILs were co-cultured with irradiated feeder cells, 3000 IU/mL IL-2, 10 ng/mL IL-21, and anti-dog CD3. Data show the total fold change in TILs over the course of the REP. **(F)** Matched quantification of total CD5^+^ T cell numbers between tumor digest and day 23 post-REP TILs. **(G)** Fold change in CD5^+^ T cell numbers throughout entire expansion phase. **(H-I)** Percentage of **(H)** CD8^+^ and **(I)** CD4^+^ subsets within the CD5^+^ compartment in matched tumor digests and post-REP TILs. **(J)** Fold expansion of CD4^+^ and CD8^+^ T cells throughout the entire expansion period (pre-REP and REP). Each point represents an individual dog; bars indicate mean ± SD. Statistical significance was determined by paired two-tailed t-tests. Asterisks indicate significance levels (*p < 0.05; ns, not significant).

We assessed four canine oral melanoma tumor digests for baseline T cell infiltration. On average, the proportion of CD5^+^ T cells within these digests was 4.63% (**Figure 1A**). T cells were activated with anti-dog CD3/CD28 beads in the presence of IL-2 for 13 days (pre-REP). Total live cell counts decreased in three of the four dog samples (**Figure 1B**), but the CD5^+^ population expanded selectively, resulting in enrichment within this fraction. **Supplementary Figure 1A** presents the same pre-REP expansion data as fold change relative to starting cell input. In a representative patient sample, the day 13 product was immunophenotyped to demonstrate this CD5^+^ selective enrichment (**Figures 1C, D**). Although the total number of live cells decreased from 1×10^6^ cells to 0.76×10^6^ (**Figure 1B**), the proportion of CD5^+^ cells increased from 3.79% on day 1 to 91.40% by day 13 (**Figure 1C**), and the total CD5^+^ cell number increased from 3.79e4 to 6.91e5 (**Figure 1D**). Therefore, while the total cell fold change was 0.76, the CD5^+^ cell fold change was 18.2, demonstrating selective expansion of T cells from the tumor digest. Within this day 13 product, CD8^+^ cells expanded preferentially (**Figure 1D**), and the CD8/CD4 ratio rose from 0.25 to 1.52 over the same period.

Preliminary REP expansions indicated that K562 cells engineered to express various costimulatory domains were more reliable feeder cells than canine peripheral blood mononuclear cells (PBMCs), as only 1/5 (20%) PBMC-stimulated cultures expanded well compared to 5/5 (100%) of K562-stimulated cultures (**Supplementary Figure 2A**). TILs expanded efficiently with K562 feeders at a 50:1 feeder-to-TIL ratio, with yields comparable to those typically achieved at the 200:1 ratio used in human studies, thus reducing feeder cell requirements (**Supplementary Figure 2B**). Further, we compared expansion in 3000 IU/mL IL-2 with or without 10 ng/mL IL-21. The addition of IL-21 enhanced TIL expansion without significantly altering memory profiles in total CD5^+^ T cells (**Supplementary Figures 3A, B**) or within CD4^+^ and CD8^+^ subsets (data not shown). IL-21 supplementation produced TIL products with a higher proportion of CD8^+^ T cells, a lower proportion of CD25^+^ T cells, and was accompanied by a significant reduction in CD4^+^CD25^+^Helios^+^FOXP3^+^ regulatory T cell (Treg) expansion (**Supplementary Figures 3C–E**). Therefore, we performed all subsequent REPs using K562 feeder cells at a 50:1 feeder-to-TIL ratio with IL-2 and IL-21 cytokine support for 9–10 days.

REP on four different day 13 OM TIL products using these methods resulted in robust proliferation with an average total live cell fold change of 177 ± 64 (**Figure 1E**). When considering both pre-REP and REP phases, CD5^+^ cells expanded an average of 3,682 ± 2,777-fold (**Figures 1F,G**). **Figure 1F** illustrates the absolute number of CD5^+^ cells from matched tumor digest to post-REP TILs, whereas **Supplementary Figure 1B** displays the corresponding expansion kinetics utilizing total viable cell counts. CD8^+^ T cells continued to expand preferentially during REP, with significantly higher proportions in post-REP products compared to the original tumor digests (p = 0.0203, **Figure 1H**), while CD4^+^ proportions showed a non-significant change (p = 0.2080, **Figure 1I**). Across all four samples, the CD8^+^ TIL fraction exhibited greater fold expansion than the CD4^+^ TIL fraction (p = 0.1431, **Figure 1J**).

Together, these results demonstrate that we can reliably expand TILs from canine oral melanoma tumor digests despite low starting T cell numbers. The inclusion of a pre-REP phase enriches for CD5^+^ T cells, and both pre-REP and REP stages preferentially expand CD8^+^ TILs, a subset typically associated with cytotoxic antitumor activity.

### Post-REP oral melanoma TILs are predominantly effector cells

**Figure 2:**
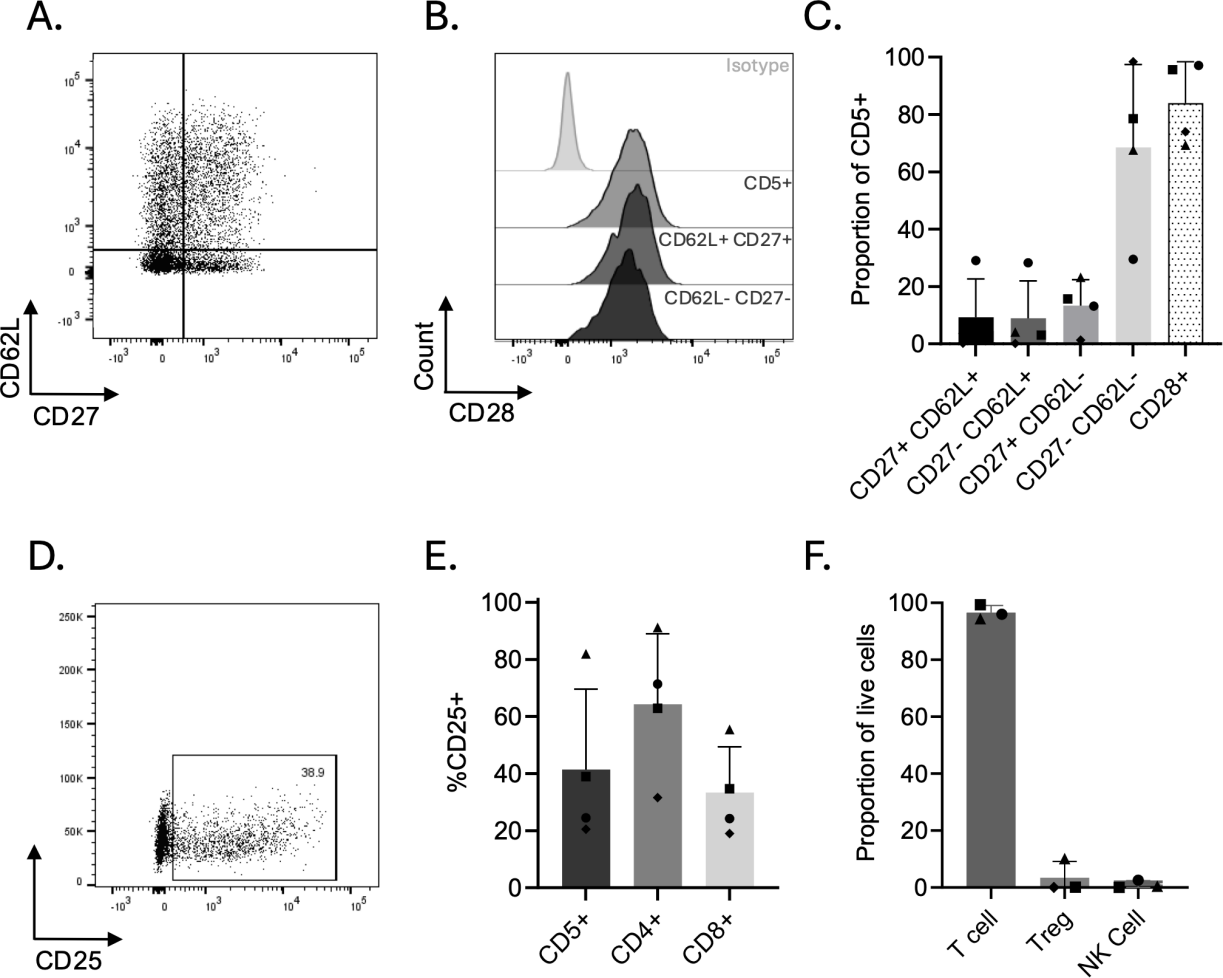
Phenotypic characterization of post-REP oral melanoma TILs. Flow cytometric analysis of expanded TILs from oral melanoma tumors. **(A)** Representative flow staining of memory markers CD27 and CD62L on CD5^+^ post-REP TILs from one dog. **(B)** Representative flow staining of costimulatory receptor CD28 expression on total T cells (CD5^+^), less differentiated T cell subsets (naïve and central memory-like; CD5^+^CD62L^+^CD27^+^), and more differentiated T cell subsets (effector-like; CD5^+^CD62L^-^CD27^-^) within post-REP TIL product from one dog. **(C)** Proportion of memory-associated and costimulatory phenotypes and in CD5^+^ post-REP TILs (n = 4). **(D)** Representative flow staining of activation marker CD25 expression on CD5^+^ post-REP TILs from one dog. **(E)** Activation marker CD25 expression within bulk CD5^+^, CD4^+^, and CD8^+^ subsets (n = 4). **(F)** Proportion of CD5^+^ T cells, CD4^+^CD25^+^Helios^+^FOXP3^+^ regulatory T cells (Tregs), and CD3^-^CD94^+^ natural killer (NK) cells within live post-REP TIL products (n = 3). Each point represents an individual dog; proportions are represented as the mean ± SD.

We evaluated memory markers on post-REP OM TIL products and immunophenotyped by flow cytometric analysis (**Figures 2A–C**), with gating strategies outlined in **Supplementary Figure 4**. Across four dogs, the majority of expanded TILs exhibited an effector-like phenotype characterized by loss of CD62L and CD27 expression (CD62L^-^CD27^-^; 68.50% ± 28.96%; **Figure 2C**). One dog had a less differentiated post-REP TIL phenotype, with 57.4% of the TIL product retaining CD62L expression, a marker typically found in naïve and central memory T cell compartments (**Figures 2A,C**). To assess CD28 expression and determine whether it was higher in less differentiated T cell compartments, we examined CD28 expression across phenotypically defined subsets in this less differentiated post-REP TIL product. Considering the incomplete characterization of canine T cell differentiation hierarchies, subsets were broadly defined using established human T cell phenotypic analogs, and CD28 expression was compared between less differentiated (naïve and central memory-like; CD62L^+^CD27^+^) and more differentiated (effector-like; CD62L^-^CD27^-^) populations within the CD5^+^ subset, relative to the expression in the overall CD5^+^ population (**Figure 2B**). Although a slight decrease in CD28 mean fluorescence intensity was observed in the CD62L^-^CD27^-^ subset, CD28 expression remained consistently high in both populations, indicating widespread preservation of CD28 across differentiation states within the expanded TIL product. Given the overall similarity in CD28 expression between subsets, subsequent analyses were performed on bulk CD5^+^ T cells. Expanded TILs consistently maintained CD28 expression across products (84.00% ± 14.40% CD28^+^; **Figure 2C**), suggesting preserved costimulatory capacity.

We evaluated CD25 expression to investigate the activation status of the post-REP TIL products. CD25 was detectable on post-REP CD5^+^ TILs but showed substantial inter-patient variability (range 20.50%–81.90%; **Figures 2D, E**). In all four patients, CD25 was expressed in a greater proportion of the CD4^+^ subset than the CD8^+^ subset. However, in three of the four patients, CD8^+^ T cells predominated, so the overall CD25 profile of the CD5^+^ population reflected that of the CD8^+^ compartment. In the remaining patient, where CD4^+^ cells outnumbered CD8^+^ cells, global CD25 expression was correspondingly higher (**Figure 2E**). To determine whether Tregs contribute to these CD4^+^CD25^+^ populations, we assessed Helios and FOXP3 expression and detected Helios^+^FOXP3^+^ Tregs only in the dog with the most robust CD4^+^ compartment, comprising 10.1% of the total live cell population (**Figure 2F**). We further assessed CD3^-^ CD94^+^ natural killer (NK) cells to confirm that our expansion protocol predominantly expanded T cells rather than NK cells (**Figure 2F**).

Overall, these data suggest that TILs expanded from canine oral melanoma tumors mainly develop effector-like phenotypes, maintain their costimulatory potential, are distinct from NK populations, and exhibit patient-dependent heterogeneity in activation status and level of Treg composition.

### Post-REP oral melanoma TILs are functional *in vitro* and largely autoreactive against tumor cells

To determine whether post-REP TILs could recognize autologous tumor cells, we co-cultured TILs with matched primary tumor digests and measured IFN-γ production in the supernatant. Because tumor digests were limited, we did not deplete CD45^+^ cells prior to reactivity assays but included tumor-alone controls to account for possible background IFN-γ production within digests. A baseline level of IFN-γ production was also observed in some TIL-only conditions, consistent with the activated state of these cells following expansion in high-dose IL-2. Accordingly, reactivity was defined as IFN-γ production above baseline, using the higher of TIL-only or tumor-only conditions as the reference for each sample.

**Figure 3:**
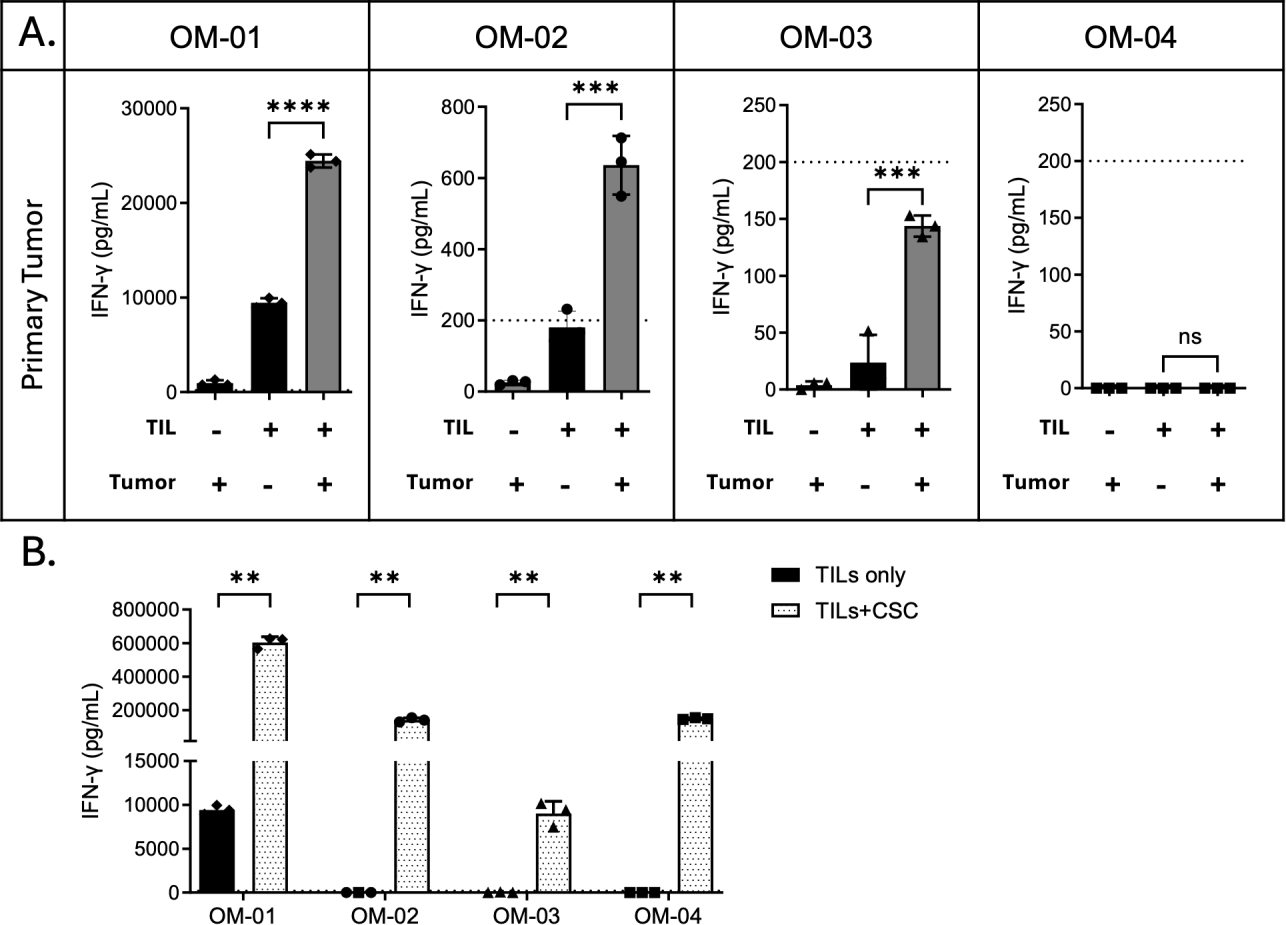
TILs expanded from primary canine oral melanoma are largely autoreactive toward autologous tumor cells. **(A)** Post-REP TILs were co-cultured with autologous tumor digests at a 1:1 effector-to-target ratio for 24 hours. Media supernatants were collected, and IFN-γ secretion was measured by ELISA. Experiments were performed in triplicate wells per condition for each dog. Individual dogs are reported separately (n = 4). Statistically significant differences within each dog were determined by one-way ANOVA with Tukey’s multiple comparisons test. **(B)** 1 × 10^5^ post-REP TILs were seeded per well in 96-well plates and treated with or without cell stimulation cocktail (CSC) for 24 hours. IFN-γ secretion was measured by ELISA. Experiments were performed in triplicate wells per condition for each dog (n = 4), and results are reported as mean ± SD of technical triplicates. Multiple paired t-tests were performed, and p-values were adjusted for multiple comparisons using BKY FDR correction. Asterisks indicate significance levels (**p < 0.01; ***p < 0.001; ****p < 0.0001; ns, not significant).

Two of the four OM TIL products generated over 200 pg/mL IFN-γ when co-cultured with autologous tumor digests, a threshold commonly regarded as baseline reactivity. These responses were also significantly greater than TILs cultured alone (p < 0.05), indicating robust autologous reactivity (OM-01 and OM-02; **Figure 3A**). A third product showed significantly elevated IFN-γ compared to background (OM-03; p = 0.0002; **Figure 3A**), but levels remained below the 200 pg/mL cutoff, suggestive of low-level reactivity. The remaining product showed no evidence of reactivity by either metric (OM-04; **Figure 3A**). All four TIL products were able to produce high levels of IFN-γ upon stimulation with cell stimulation cocktail (CSC), suggesting that the lack of reactivity in some products was not due to functional anergy but rather to a lack of sufficient tumor-specific recognition (**Figure 3B**).

### Oral melanoma TIL functionality is DLA-dependent

To determine whether IFN-γ production is dog leukocyte antigen (DLA)-dependent, we tested reactivity against a parental and β2-microglobulin (B2M) knockout (KO) cell line generated from the primary melanoma tumor digest that produced the most robust TIL IFN-γ response (OM-01; **Table 1**).

**Table 1:** Autologous reactivity across all canine TIL samples.

Patient ID	TIL expansion	Primary tumor reactivity			Cultured tumor reactivity		
	CD5^+^ fold change	Background IFN-γ (pg/mL)	Tumor + TIL IFN-γ (pg/mL)	Statistical significance	Background IFN-γ (pg/mL)	Tumor + TIL IFN-γ (pg/mL)	Statistical significance
OM-01	7055	9,428	24,432	****	9,428	15,884	****
OM-02	1195	180	636	***	218	390	*
OM-03	1628	24	144	***	N/A		
OM-04	4848	0	0	ns	N/A		
OSA-01	6674	1,451	3,466	***	1,783	6240	****
OSA-02	3809	N/A			856	3146	***
OSA-03	184	46	458	****	239	390	****
OSA-04	432	108	730	****	N/A		
OSA-05	2733	5	255	****	5	153	**
OSA-06	568	291	685	***	291	176	ns
OSA-07	548	34	386	***	34	122	**
OSA-08	2675	N/A			59	144	*
OSA-09	7350	44	132	**	44	136	*
OSA-10	4424	50	79	ns	51	40	ns
OSA-11	2266	22	48	ns	N/A		

IFN-γ concentrations are reported as the mean of technical triplicates. Background IFN-γ concentrations were defined as the higher value measured in tumor-only or TIL-only cultures. Statistical significance was assessed using one-way ANOVA with Tukey’s multiple comparisons test. Asterisks indicate significance levels (*p < 0.05; **p < 0.01; ***p < 0.001; ****p < 0.0001; ns, not significant). N/A indicates that the assay was not performed (e.g., insufficient tumor sample). OM, oral melanoma; OSA, osteosarcoma.

**Figure 4:**
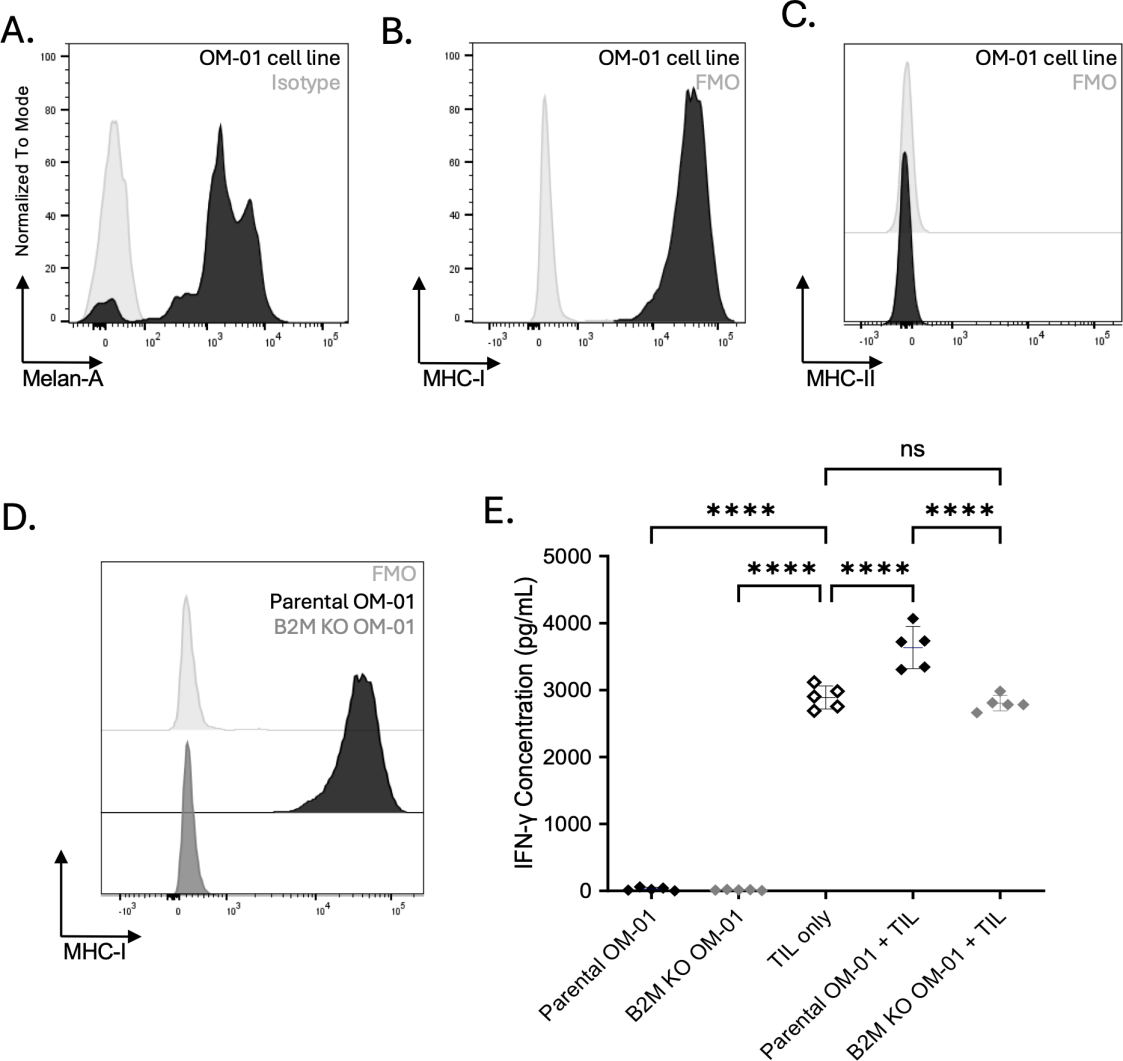
Melanoma OM-01 TIL reactivity is DLA-dependent and can be maintained against autologous tumor cell lines. **(A-C)** Flow cytometric characterization of the patient-derived tumor cell line for intracellular Melan-A expression **(A)**, MHC class I **(B)**, and MHC class II **(C)** surface expression. **(D)** β2-microglobulin (B2M) was disrupted in the parental tumor cell line using CRISPR/Cas9, and loss of MHC class I was confirmed by flow cytometry. **(E)** Post-REP TILs were co-cultured with the parental or B2M knockout autologous tumor cell line for 24 h. IFN-γ secretion was measured in supernatants by ELISA. Reactivity was maintained against the parental line but abolished following MHC class I loss. Results are reported as mean ± SD of technical replicates. Statistical significance was determined by one-way ANOVA with Tukey’s multiple comparisons test. Asterisks indicate significance levels (****p < 0.0001; ns, not significant).

We established an early-passage parental canine oral melanoma cell line from this tumor digest and confirmed expression of the melanocytic differentiation marker Melan-A (**Figure 4A**). Flow cytometric analysis showed strong expression of MHC class I, with negligible class II expression (**Figures 4B,C**). To disrupt class I antigen presentation, we edited B2M, a molecule necessary for stable class I surface expression, using CRISPR/Cas9. The loss of MHC class I expression in the B2M KO line confirmed successful gene disruption (**Figure 4D**).

Reactivity assays demonstrated that patient-matched TILs produced an IFN-γ response only when co-cultured with the parental tumor cell line but not when co-cultured with the isogenic B2M KO line (**Figure 4E**). Notably, IFN-γ levels in the B2M KO co-culture dropped to baseline levels produced by unstimulated TIL controls. These results suggest that autologous reactivity of canine oral melanoma TILs is dependent on the presentation of tumor antigens by MHC class I.

### TILs can be expanded from canine appendicular osteosarcoma tumor digests

**Figure 5:**
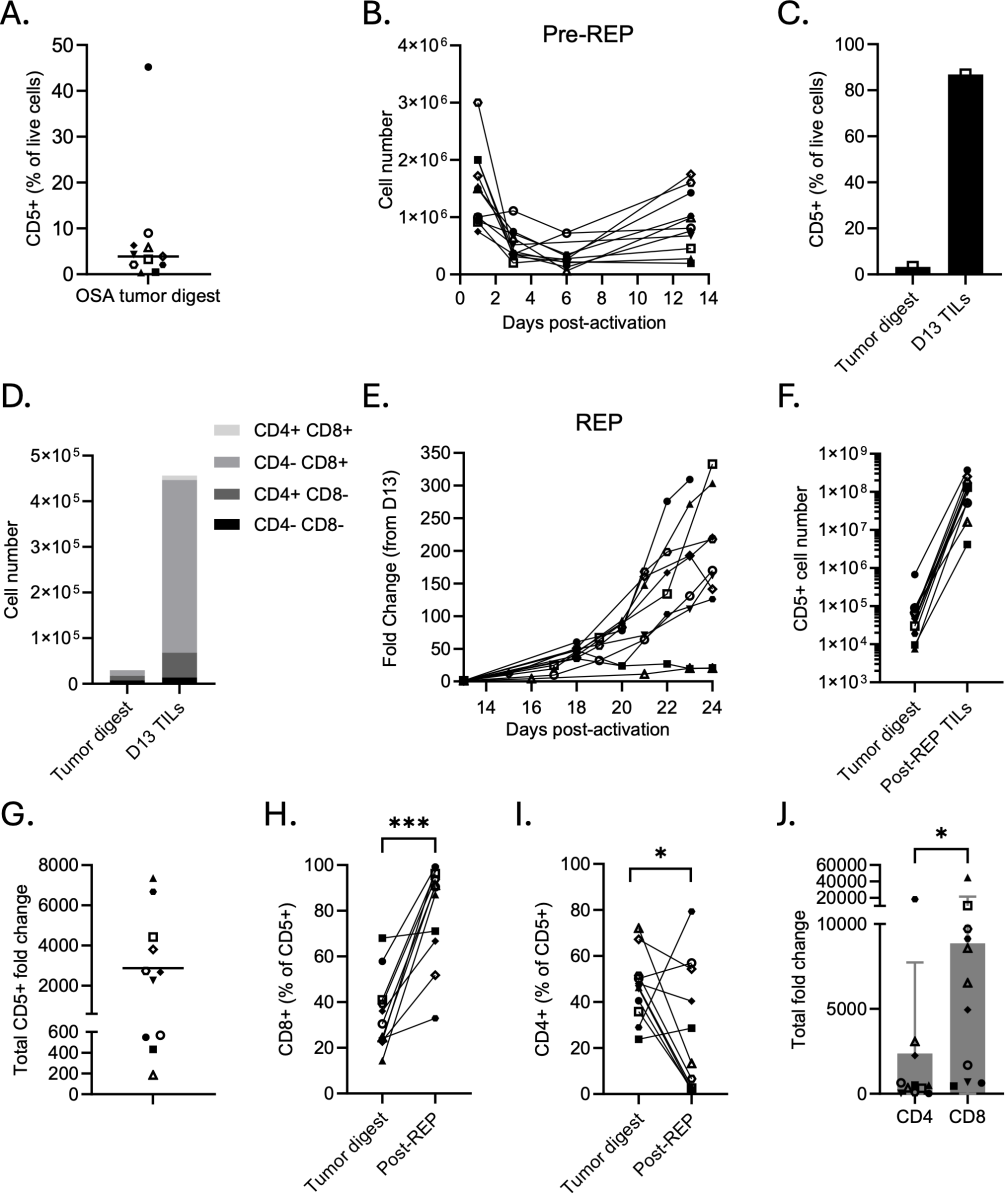
TILs can be expanded from primary canine osteosarcoma tumors. **(A)** Flow cytometric analysis of primary tumor digests to determine the starting percentage of CD5^+^ T cells (n = 11). **(B)** Change in total cell number from oral melanoma tumor digests (day 1) during the pre-REP phase. TILs within the digests were activated with anti-dog CD3/CD28 beads in the presence of 3000 IU/mL IL-2 for 12 days. **(C)** Representative flow cytometric comparison of tumor digest and day 13 (pre-REP) TILs, demonstrating enrichment of CD5^+^ T cells. **(D)** Representative analysis of CD5^+^CD4^+^ and CD5^+^CD8^+^ subsets. **(E)** Growth kinetics of TILs during REP. Pre-REP TILs were co-cultured with irradiated feeder cells, 3000 IU/mL IL-2, 10 ng/mL IL-21, and anti-dog CD3. Data represent the total fold change in TILs over the course of the REP. **(F)** Matched quantification of total CD5^+^ T cell numbers between tumor digest and post-REP TILs. **(G)** Fold change in CD5^+^ T cell numbers throughout entire expansion phase. **(H-I)** Percentage of **(H)** CD8^+^ and **(I)** CD4^+^ subsets within the CD5+ compartment in matched tumor digests and post-REP TILs. **(J)** Fold expansion of CD4^+^ and CD8^+^ T cells throughout the entire expansion period (pre-REP and REP). Each point represents an individual dog; bars indicate mean ± SD. Statistical significance was determined by paired two-tailed t-tests. Asterisks indicate significance levels (*p < 0.05, ***p < 0.001).

To determine whether TIL expandability and functionality extend beyond melanoma, we examined tumor digests from canine appendicular osteosarcoma. We digested eleven OSA tumors into single-cell suspensions and analyzed them for T cell content, which varied widely across samples (range 0.38%–45.20%; mean 7.52%; **Figure 5A**). 8/11 (72.7%) OSA TIL products demonstrated an overall reduction in live cell numbers during the pre-REP phase (**Figure 5B**), with fold changes depicted in **Supplementary Figure 1C**. However, we observed selective enrichment of CD5^+^ T cells during this phase, demonstrated by a representative patient, where the proportion of CD5^+^ cells increased from 3.26% on day 1 to 86.9% by day 13 (**Figure 5C**), despite a decrease in overall live cell number (**Figure 5B**). This enrichment was accompanied by preferential expansion of CD8^+^ T cells, with the CD8/CD4 ratio rising from 1.15 to 6.08 (**Figure 5D**).

Across 11 samples, OSA TILs exhibited variable proliferation among patients, with an average REP fold expansion of 184 ± 107 and an average CD5^+^ fold expansion over the entire expansion period of 2,878 ± 2,492 (**Figures 5E–G**). **Figure 5F** shows the absolute CD5^+^ cell number from matched tumor digest to post-REP TILs, while **Supplementary Figure 1D** presents the same expansion kinetics using total viable cell numbers. When comparing tumor digest to post-REP products, CD8^+^ T cell proportions increased across all patients (p = 0.0001; **Figure 5H**), whereas CD4^+^ proportions tended to decrease, though not uniformly (p = 0.0418; **Figure 5I**). CD8^+^ TILs exhibited significantly greater fold expansion than CD4^+^ TILs, with average fold changes of 8,861 ± 12,521 and 2,372 ± 5,367 in CD8^+^ and CD4^+^ TILs, respectively (p = 0.0322; **Figure 5J**). Overall, TILs from canine appendicular osteosarcoma tumor digests can be expanded, even in contexts with low baseline T cell infiltration.

### Post-REP OSA TILs are predominantly effector cells

**Figure 6:**
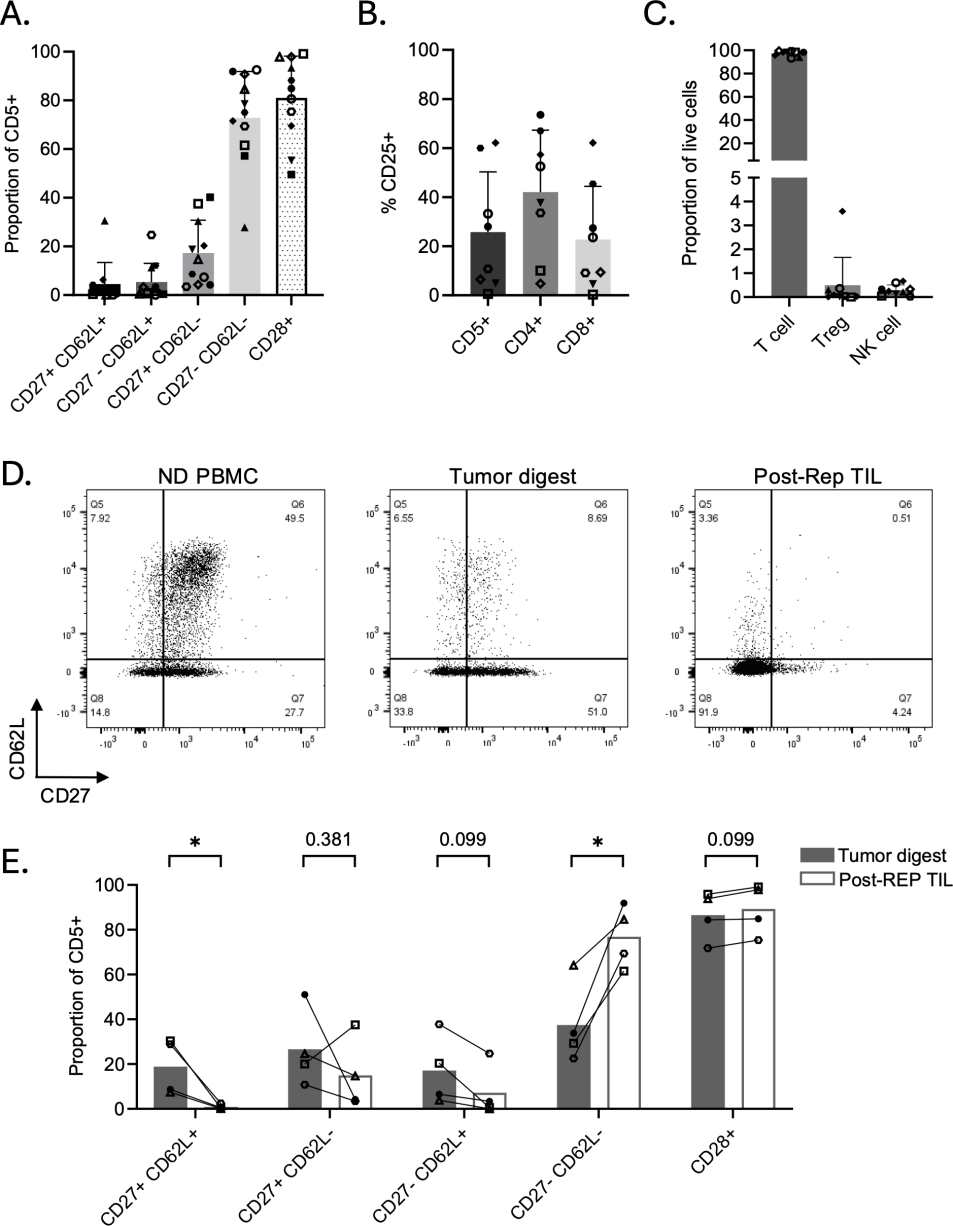
Phenotypic characterization of post-REP osteosarcoma TILs. Flow cytometric analysis of expanded CD5^+^ TILs from osteosarcoma tumors. **(A)** Proportion of memory-associated and costimulatory phenotypes and in CD5^+^ post-REP TILs (n = 11). **(B)** Activation marker CD25 expression within bulk CD5^+^, CD4^+^, and CD8^+^ subsets (n = 8). **(C)** Proportion of CD5^+^ T cells, CD4^+^CD25^+^Helios^+^FOXP3^+^ regulatory T cells, and CD3^-^CD94^+^ natural killer cells within live post-REP TIL products (n = 9). Proportions are represented as the mean ± SD. **(D)** Representative flow plots of memory subsets in normal donor (ND) PBMCs compared to tumor digest and post-REP TILs from one dog. **(E)** Comparison of memory-associated phenotypes between tumor digest and post-REP TILs defined by CD27, CD62L, and CD28 expression (n = 4). Multiple paired t-tests were performed, and p-values were adjusted for multiple comparisons using the BKY two-stage FDR method. Each point represents an individual dog; proportions are represented as the mean ± SD. Asterisks indicate significance levels (*p < 0.05).

Similar to OM, phenotypic analysis of post-REP OSA-derived TILs showed a profile consistent with an effector-like state, with the majority of cells lacking CD62L and CD27 expression (72.78% ± 19.13% CD62L^-^CD27^-^; **Figure 6A**). These TILs largely maintained CD28 expression (81.07% ± 17.12%; **Figure 6A**), indicating preserved co-stimulatory ability. CD25 expression varied among samples and was more pronounced within CD4^+^ subsets in 6/8 products tested (**Figure 6B**). The post-REP product with the highest global CD25 expression was the only product that had a measurable Helios^+^FOXP3^+^ Treg population, at 3.59% of live cells, and no products had an NK cell population (n=9; **Figure 6C**).

To evaluate how ex vivo expansion altered T cell phenotype, we compared paired samples from tumor digests and matched post-REP TILs in four dogs. Representative staining of CD62L and CD27 in matched tumor digest and TILs from one dog compared to normal donor PBMCs suggests that T cells within digests are more differentiated than healthy donor circulating PBMCs, but less so than expanded TIL products (**Figure 6D**). When pooling the proportion of these memory subsets across four dogs, we observed a significant reduction in less differentiated CD62L^+^CD27^+^ fractions (p = 0.0481; **Figure 6E**), likely harboring the naïve and central memory subsets, and a significant increase in the CD62L^-^CD27^-^ effector states in TIL products compared to matched tumor digests (p = 0.0481; **Figure 6E**). CD28 expression was maintained throughout ex vivo expansion in all four samples (**Figure 6E**). These data suggest that TIL expansion supports differentiation toward a more effector-like phenotype compared to freshly isolated TILs.

### Post-REP OSA TILs are functional *in vitro* and largely autoreactive against tumor cells

To evaluate the tumor reactivity of OSA-derived TILs, we conducted IFN-γ release assays by co-culturing post-REP TILs with matched primary tumor digests, summarized in **Table 1**. Out of nine OSA TIL products with sufficient tumor digest available, six (66.7%) demonstrated robust autologous reactivity, producing over 200 pg/mL of IFN-γ in response to tumor co-culture and significantly higher levels compared to tumor or TILs cultured alone (p < 0.05). One product generated IFN-γ levels above background but below the 200 pg/mL threshold, indicating low-level reactivity. The remaining two products showed no detectable reactivity. All OSA-derived TILs maintained the ability to produce high levels of IFN-γ when stimulated with cell stimulation cocktail, suggesting that lack of reactivity was due to an absence of tumor-specific recognition rather than global T cell dysfunction (**Supplementary Figure 5**).

**Figure 7:**
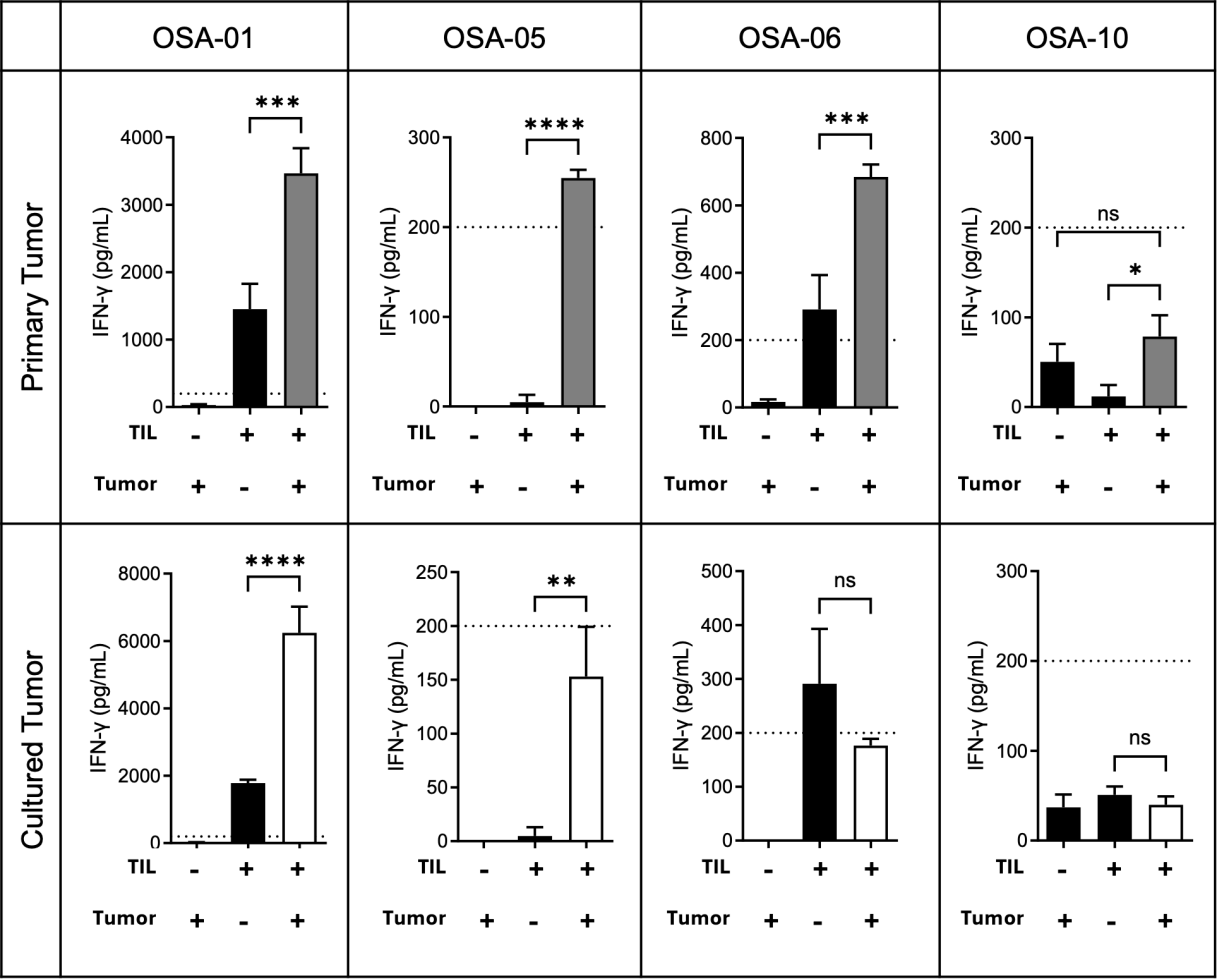
Autologous reactivity of osteosarcoma TILs against primary tumor digests and early passage tumor cell lines. Post-REP TILs were co-cultured with either autologous primary tumor digest or early passage autologous tumor cell lines at a 1:1 effector-to-target ratio for 24 hours. IFN-γ secretion was quantified by ELISA. Black bars represent TILs or tumor cultured alone, gray bars represent TILs cultured with primary tumor digest, and open bars represent TILs cultured with early passage tumor cell lines. Data are shown for four representative patients; additional OSA patients are summarized in **Table 1**. Strong reactivity was defined as IFN-γ production greater than 200 pg/mL and significantly higher than that of TILs and tumor cultured alone (p < 0.05). Results are reported as mean ± SD of technical triplicates. Statistical significance was assessed using one-way ANOVA with Tukey’s multiple comparisons test. Asterisks indicate significance levels (*p<0.05, **p < 0.01, ***p < 0.001, ****p < 0.0001; ns, not significant).

Next, we explored whether early passage tumor cell lines could serve as a substitute for autologous tumor digests in reactivity assays when tumor samples are scarce. We successfully generated early passage lines from nine OSA tumors, seven of which had matched tumor digest co-cultures, and tested them against patient-matched TILs. In 2/7 (28.6%) of these cases, robust reactivity against the primary tumor digest was preserved when tested against the corresponding cell line, indicating retention of relevant antigens, as shown for a representative patient (OSA-01; **Figure 7**) and summarized in **Table 1**. In other cases, reactivity against the corresponding cell line was reduced or absent; specifically, IFN-γ production fell below 200 pg/mL in 3/7 (42.9%) samples and was completely lost in 1/7 (14.3%) as shown for representative patients (OSA-05 and OSA-06; **Figure 7**), suggesting changes in antigen expression or cell composition during ex vivo culture. As anticipated, the 1/7 (14.3%) TIL products that did not react to primary tumor digests also failed to respond to the corresponding cell line (OSA-10; **Figure 7**). For two patients with tumor digest co-cultures, matched early passage cell lines could not be established. Overall, 9/11 (81.8%) canine OSA TIL products demonstrated IFN-γ responses upon co-culture with autologous tumor cells (either primary or cultured). Of these, 7/9 (77.8%) produced greater than 200pg/mL of IFN-γ, indicating robust autologous reactivity. These results are summarized in **Table 1**.

### Associations between TIL expansion and reactivity

**Figure 8:**
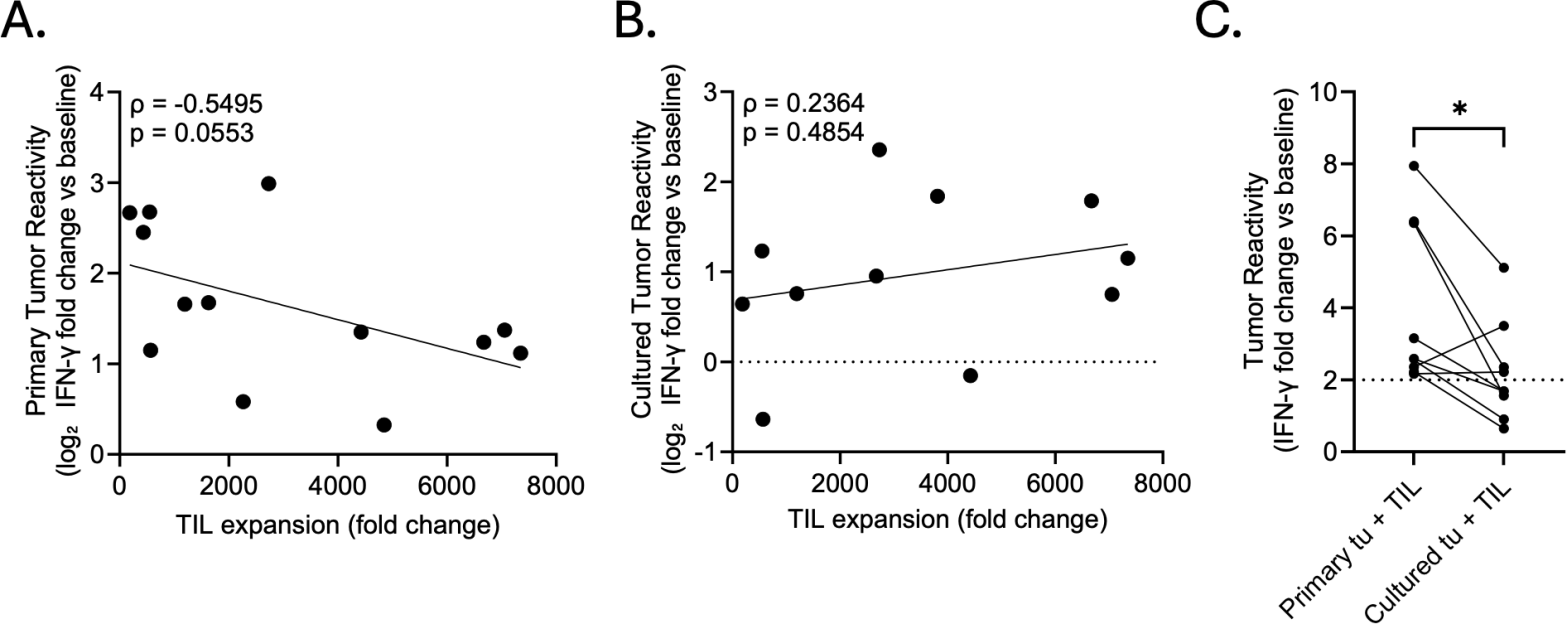
Associations between TIL expansion and autologous reactivity. (**A, B**) Tumor reactivity was quantified as the log_2_ fold change in IFN-γ secretion by expanded TILs following co-culture with autologous **(A)** primary tumor (n = 13) and **(B)** early passage tumor cell lines (n = 11) relative to baseline. TIL expansion is shown as fold change of TILs during the entire expansion period. Each point represents an individual TIL product. Melanoma and osteosarcoma patients are combined. Spearman’s rank correlation coefficient (ρ) and associated p-value are displayed. **(C)** Matched tumor reactivity of TILs tested against primary tumor digest and early passage tumor cell lines derived from the same patient (n = 9). Statistical significance was assessed using a paired Wilcoxon signed-rank test. Asterisks indicate significance levels (*p < 0.05).

To assess whether the proliferative capacity of TILs correlates with their functional activity, we compared total T cell fold expansion to autologous reactivity, including both OM and OSA products. Tumor reactivity was quantified as the log_2_ fold change in IFN-γ secretion measured during co-culture with autologous tumor relative to baseline TIL culture. We observed a moderate inverse association between TIL expansion and primary tumor reactivity (Spearman ρ = –0.5495, p = 0.0553; *n* = 13; **Figure 8A**), trending toward statistical significance. However, this association flipped when TIL expansion was compared to reactivity against cultured tumor cells, which resulted in a weak positive association (Spearman ρ = 0.2364, p = 0.4854; *n* = 11; **Figure 8B**), though not statistically significant. For the nine patients with both sufficient primary tumor digest and an early passage autologous tumor cell line (2 OM, 7 OSA), we directly compared matched reactivity assays. Patient-matched TIL products demonstrated reduced reactivity toward early tumor culture compared to fresh tumor digests (p = 0.0195, **Figure 8C**).

In parallel, we assessed the impact of sample collection and processing speed on tumor digest viability and whether sample viability influenced TIL expansion or function. Post-processing viability was compared between freshly processed and overnight-shipped samples, and correlated with both TIL fold expansion and autologous primary tumor reactivity. While shipped samples exhibited a subset with reduced viability, no meaningful correlation was observed between viability and TIL expansion, indicating that lower viability samples were still capable of generating robustly expandable TIL products (Spearman ρ = 0.1270, p = 0.6499; *n* = 15; **Supplementary Figures 6A, B**). A weak, non-significant positive association was observed between viability and primary tumor reactivity (Spearman ρ = 0.3471, p = 0.2438; *n* = 13; **Supplementary Figure 6C**), which may reflect greater preservation of viable T cells and, consequently, a broader repertoire of tumor-reactive clones; however, this interpretation remains speculative.

## Discussion

Tumor immunogenicity is linked to the mutational landscape. Mutational burden correlates with neoantigen load, and multiple studies show that both measures correlate with overall and progression-free survival following TIL adoptive transfer (5, 9, 10, 31). This relationship helps elucidate why human cutaneous melanomas, largely driven by UV-mutagenesis, respond favorably to TIL therapy (2). In contrast, tumors with intrinsically low TMB, such as mucosal melanomas and sarcomas, are generally considered less immunogenic and more resistant to immunotherapies (e.g., immune checkpoint inhibition) (32). However, in the C-144–01 lifileucel trial, a small subset of 12 mucosal melanoma patients achieved an ORR of 50%, suggesting that even low-TMB tumors can respond to TIL therapy under favorable conditions (25). These findings emphasize that neoantigen abundance is not the sole determinant of TIL therapeutic efficacy and must be evaluated alongside other features, such as T-cell fitness, cytokine milieu, and the tumor microenvironment. This complexity in determining response highlights the need for robust preclinical models.

Approximately 92% of drugs deemed safe and effective in conventional animal models fail to translate successfully to humans (33). Dogs, unlike traditional murine models, are uniquely positioned as comparable patient populations that could reduce this failure rate due to their closer similarity to humans. This similarity stems, in part, from shared environmental exposures, spontaneous disease development, genetic diversity, and competent immune systems (34). Additionally, unlike smaller rodents, their size allows for a more accurate assessment of drug doses, pharmacodynamics/pharmacokinetics, and biodistribution profiles (35). Clinical trials in pet dogs also offer unique advantages over human trials, such as fewer regulatory restrictions and accelerated clinical outcomes due to shortened lifespan and rapid disease progression (34). As a result, canine cancers present a valuable and underused resource in cancer therapy research and serve as an ideal model to dissect TIL biology and explore determinants of response.

Canine oral melanoma and osteosarcoma share overlapping molecular pathways and clinical features with human mucosal melanoma and pediatric OSA, respectively (36, 37). Despite their low TMB-status, immune infiltration patterns observed across both tumor types and species have prognostic value (29, 38), suggesting that immune cell composition is biologically and clinically relevant in these cancers. Our results indicate that canine TILs can be successfully expanded from both OM and OSA tumors, achieving sufficient yields for clinical application. In key human studies, rapid expansion protocols (REPs) typically generate ~1,000-fold increase in melanoma TIL numbers, with lower fold expansion reported for sarcomas (39–42). By comparison, our REP-only phase achieved mean expansions of 177-fold for OM and 184-fold for OSA. The lower expansion may be attributable, in part, to the shorter expansion duration, as our 9–10-day REP is shorter than the 12–14-day REP commonly used in human TIL protocols (43). Additionally, compared with human systems, canine T cell expansion is constrained by the limited availability of canine-specific reagents optimized for ex vivo expansion – including a lack of commercially available activating beads, canine-specific antibodies, or canine-specific media formulations. However, when combined with a pre-REP activation stage, TIL expansion can reach clinically meaningful levels in the dog, boosting mean total CD5^+^ cell expansion to 3,682 fold for OM and 2,878-fold for OSA in our study. Considering tumor size, all but one product in our study was expected to exceed 10^9^ total T cells, a clinically relevant threshold for therapeutic infusion (44). These results highlight the feasibility of producing large-scale TIL products from canine tumors despite low baseline intratumoral T-cell infiltration.

The relative contribution of human CD8^+^ and CD4^+^ T-cell subsets to TIL efficacy remains only partly understood (45). CD8^+^ T cells have long been regarded as the primary mediators of tumor rejection due to their cytolytic capacity, but growing evidence indicates that CD4^+^ T cells play essential cytokine and helper roles that enhance TIL expansion, persistence, and function (46). Several human TIL studies have correlated clinical responses to the total number of infused CD8^+^ cells, highlighting the importance of this subset, although CD4^+^ cells still play a key role in modulating the immune response (10, 47, 48). In our canine TIL products, CD8^+^ cell populations largely predominated after REP, while CD4^+^ levels varied among patients. This variability offers a chance to explore how the balance between these subsets influences tumor recognition and persistence in an immune-competent environment.

Post-REP TILs from both OM and OSA exhibited predominantly effector-like phenotypes (CD62L^-^), suggesting prior activation and differentiation (49–51). While ex vivo expansion further pushes differentiation, T cells within matched tumor digests were skewed toward CD62L^-^ subsets at baseline, likely reflecting chronic antigen exposure within the tumor microenvironment prior to tumor resection. Canine post-REP TILs largely retain CD28 expression, a feature reported in effector memory T cells and thought to be lost primarily during terminal stages of T cell differentiation in both humans and dogs (52). T effector memory that re-express CD45RA (TEMRA) status via CD45RA expression could not be directly assessed in this study due to the lack of compatible conjugated antibodies and cross-reactivity of secondary antibodies with the anti-CD3 reagent used for expansion. CD28 retention therefore suggests that expanded TILs primarily consist of effector memory cells (T_EM_; CD62L^-^CD28^+/-^CD27^+/-^), with a smaller subset of terminally differentiated effector cells (T_EFF;_ CD62L^-^CD28^-^CD27^-^) (52). The inclusion of CD27 in these subsets is based on human T cell differentiation schemes (53), as CD27 is not well characterized in canine T cells. However, we did observe high levels of CD27 in PBMCs with a reduction in highly differentiated TIL subsets, suggesting it may follow a comparable differentiation trajectory to humans. Retention of CD28 also indicates preserved costimulatory capacity, and the presence of an effector memory-like subset implies a potential for protective memory formation, tissue trafficking, and rapid effector responses (52). These canine TIL phenotypes align with human data showing that feeder-based REPs promote differentiation toward effector and effector-memory cell states. For instance, one study found that TILs expanded with anti-CD3 and IL-2 showed decreased CD62L (35%→5%) and CD27 (67%→34%) expression compared to IL-2 alone, while CD28 expression remained stable (~62%) (50). Although differentiation supports antigen recognition and antitumor responses, terminal differentiation can lead to senescence and exhaustion (10).

Further, the absence of a strong central memory compartment (T_CM_; CD62L^+^CD28^+^CD27^+^) might limit long-term memory formation and persistence. In a study tracking melanoma-reactive CD8^+^ T cells after ACT, the CD28^+^CD27^-^ population was generally short-lived and experienced significant contraction, while the CD28^+^CD27^+^ population showed greater stability (54). While CD27 and CD28 are preferentially expressed in “young” TIL and correlated with clinical responses in melanoma, reports conflict on the clinical significance of CD27 subsets, with some studies finding better outcomes for CD27^-^ T_EFF_ TILs versus CD27^+^ T_EM_ TILs (48, 55, 56). Despite this uncertainty, sustaining CD62L and CD27 expression to expand the central memory compartment is generally considered beneficial for enhancing TIL persistence *in vivo* and should be explored when further optimizing canine TIL products (12, 57–59).

Despite limited antibody availability for canine immunophenotyping, we evaluated TIL activation through expression of CD25, the *α* chain of the high-affinity IL-2 receptor. CD25 was variably expressed across patients and was generally higher within CD4^+^ subsets. While transient CD25 upregulation reflects activation and IL-2 responsiveness, persistent expression can also indicate the presence of Tregs (60). Since most canine TIL products were CD8 dominant, elevated CD25 expression likely represents activation rather than Treg expansion; however, CD4-biased products may contain regulatory subsets. Two of twelve products tested had Tregs within this CD4^+^CD25^+^ compartment, with 3.6% and 10.1% of CD5^+^ cells dually expressing Helios and FOXP3. Since both products exhibited low-level autologous reactivity, Treg presence may limit TIL anti-tumor activity in these dogs (61). However, Treg outgrowth was not a consistent finding.

One of the main challenges that restricts tumor control and long-lasting remissions after ACT is limited T cell persistence. Historically, TIL expansions used high doses of IL-2 (up to 6000 IU/mL) to promote T cell activation and growth, but such methods often push differentiation and support the growth of suppressive Tregs – factors that can contribute to limited persistence and anti-tumor activity *in vivo (*62). However, modifying the cytokine environment within cultures can alter T cell phenotypes. For example, IL-21 has been shown to play a key role in maintaining memory CD8^+^ populations, suppressing Treg outgrowth, and enhancing the expansion of CD27^+^CD28^+^ “young” TILs with superior cytotoxic capabilities (62–65). Therefore, we tested expansion protocols utilizing a lower IL-2 concentration (3000 IU/mL) with the addition of IL-21 during REP. While IL-21 improved overall canine TIL proliferation, promoted CD8^+^ expansion, and reduced Treg expansion compared to IL-2 alone, its effect on memory profiles was negligible. Introducing IL-21 or other homeostatic cytokines during the pre-REP phase or further lowering IL-2 may better preserve less-differentiated states—methods that should be explored for further canine TIL optimization. Similarly, modulating activation strength through adjustments in feeder-to-TIL ratios, reduction in culture duration, or omission of pre-REP bead activation are alternative approaches to reduce excessive differentiation, aiming to preserve “young” TIL phenotypes (66). However, such modifications must be balanced against potential yield reductions.

A significant barrier to the translation of TIL-based immunotherapies into the veterinary sector includes the limited availability of the biologics required to manufacture and study these therapies. Canine tumor biobanks are comparatively limited, and this work required our samples to be obtained fresh – heavily dependent on caseload and access – rather than pulling from pre-established banks. Consequently, we were restricted in the number and size of the digests received, constraining our analysis of tumor digests and matched TILs. Another challenge is the relative difficulty in acquiring sufficient quantities of PBMCs required for traditional PBMC-based feeder protocols. Clinical-scale TIL expansions require up to 10 billion irradiated PBMCs as feeder cells per patient, typically pooled from at least three donors (67). To overcome this limitation, we implemented an artificial antigen-presenting cell (aAPC)-based feeder system using K562 cells engineered to express various human costimulatory domains (CD80, CD83, CD86, and CD137L). These K562 cells are readily maintained *in vitro* and serve as a renewable “off-the-shelf” platform, lowering costs and reducing lot-to-lot variability compared to pooled PBMC-based feeder systems. Additionally, preliminary expansions showed more consistent TIL proliferation using our aAPCs versus canine PBMC feeders, although we couldn’t pool from multiple donors. Reducing the feeder-to-TIL ratio to 50:1 resulted in comparable expansions relative to the conventional 200:1 ratio used in human studies, thereby decreasing feeder cell requirements. Similar K562-based methods have been employed by our group and others, supporting the feasibility of this approach (66, 67).

Autologous reactivity assays confirmed that 80% of the canine TIL products generated with this approach produce IFN-γ responses when co-cultured with matched primary tumor digests or early-passage tumor cell lines. This suggests that interaction with autologous tumor antigens directly stimulates IFN-γ production and that functional capacity can be maintained *in vitro*. This pattern of reactivity reflects observations in human TIL studies, where only a portion of expanded products display tumor reactivity despite proliferative potential (39, 68). Using a strict IFN-y cutoff of 200 pg/mL, 60% of canine TIL products in our study are considered reactive by human trial standards, comparable to the 67% reported in human malignant melanoma literature (69). Notably, the frequency of strong or low-level autoreactive products in OSA is similar to the proportion observed in OM, reinforcing the broader feasibility of generating functional TILs across various canine solid tumor types. All post-REP TIL products responded to antigen-independent stimulation with a cell stimulation cocktail (PMA/ionomycin) that bypasses the TCR, indicating that functional activity is preserved after ex vivo culture. Therefore, any absence of reactivity toward autologous tumor cells is likely due in large part to a lack of antigen recognition rather than impaired functionality (70). Early passage tumor cell lines can be a useful substitute for reactivity testing when tumor digest is limited; however, results should be interpreted with caution, as reactivity is not uniformly maintained across tumor digest–cell line pairs and may underestimate the true autologous reactivity. In several cases, TILs reactive to the primary tumor exhibited reduced or absent reactivity to the corresponding cell line. Potential mechanisms for this include a decrease or alteration in cell composition or antigenic diversity in the cell line compared to the primary tumor (70, 71).

In human studies, TIL cytotoxicity is mediated by T-cell receptor recognition of antigenic peptides presented by MHC molecules, typically demonstrated by decreased IFN-γ responses in the presence of human leukocyte antigen (HLA)-blocking antibodies (67, 72). Since validated dog leukocyte antigen (DLA)-blocking antibodies are not available, we developed a cell line-based system to test MHC-dependence in canine TILs. B2M knockout in an early passage autologous tumor cell line reduced IFN-γ secretion by matched TILs down to baseline. This finding suggests that canine TIL activity is MHC-I dependent, paralleling the canonical TCR–MHC interactions observed in human TILs. Collectively, these results support the mechanistic conservation of antigen-specific recognition between species and highlight the translational value of canine models for studying class I–restricted cytotoxicity.

The presence of a moderate negative correlation between TIL proliferation and autologous reactivity is not unexpected, as all TILs were uniformly activated with CD3/CD28 beads and subsequently expanded with feeder cells, regardless of tumor-specific recognition. Under these conditions, proliferative capacity primarily reflects responsiveness to polyclonal stimulation rather than antigen specificity, augmented by the expansion of bystander non-reactive lymphocytes (10, 12). Therefore, expansion kinetics cannot serve as a biomarker for tumor reactivity, highlighting the importance of conducting *in vitro* functional assays before infusion. Rather, a wider assessment of TIL composition beyond cell yield - including differentiation, exhaustion, and costimulatory status - may be more beneficial for predicting response (10). It is notable, however, that the total and CD8^+^ infused TIL number have been shown to correlate with response to TIL therapy in both human metastatic melanoma and osteosarcoma patients, suggesting that expandability is important to reach adequate infusion numbers, and should be included in a holistic assessment of the product (10, 45, 47, 50). Nevertheless, the quality of the TIL product—defined by specificity, phenotype, and functionality—is a more critical determinant of therapeutic efficacy than the magnitude of expansion.

While this study provides a foundational framework for canine TIL therapy, it is limited to *in vitro* analyses. B2M knockout studies were performed only on tumor samples that yielded stable, expandable tumor cell lines and had matched TIL products demonstrating robust autologous reactivity to the parental line, thereby restricting the number of evaluable samples. Additionally, the limited availability of validated canine antibodies restricts in-depth immunophenotypic analysis of canine TILs by flow cytometry. Future application of single-cell sequencing and TCR profiling may enable more comprehensive assessment of canine TIL diversity and help identify molecular correlates of tumor reactivity. Future work must evaluate *in vivo* anti-tumor efficacy and pilot trials are needed to determine the safety profile in dogs, particularly when combined with systemic IL-2 administration – generally considered a necessary component in human TIL therapy but associated with substantial toxicity (73).

Collectively, this study demonstrates the feasibility of expanding, characterizing, and functionally validating TILs sourced from canine tumors. These findings lay the groundwork for future canine TIL immunotherapy trials and position dogs as a valuable comparative model to investigate determinants of TIL efficacy in low-TMB malignancies. The development of canine TIL expansion protocols enables investigation of strategies to broaden the neoantigen repertoire, such as radiation therapy, and to enhance TIL infiltration into immunosuppressive solid tumor microenvironments. By utilizing naturally occurring tumors in an immunocompetent host, this approach creates a unique translational bridge to enhance outcomes for both canine and human patients with treatment-resistant solid tumors.

## Methods

### Media and solution formulation

T cell medium (TCM) was prepared using Roswell Park Memorial Institute (RPMI) 1640 medium supplemented with GlutaMAX, 25 mM HEPES (4-(2-hydroxyethyl)-1-piperazineethanesulfonic acid), 10% heat-inactivated fetal bovine serum (FBS), 100 U/mL of penicillin, 100 μg/mL of streptomycin, and 50 µM 2-mercaptoethanol. Primary tumor cell lines were expanded in Dulbecco’s Modified Eagle Medium (DMEM) containing GlutaMAX, 4.5 g/L D-glucose, 10% heat-inactivated FBS, 100 U/mL of penicillin, and 100 μg/mL of streptomycin. RPMI complete medium (CM) was formulated with RPMI 1640 containing GlutaMAX, 25 mM HEPES, 10% heat-inactivated FBS, 100 U/mL of penicillin, and 100 μg/mL of streptomycin. Tumor transport medium was prepared with Hanks’ Balanced Salt Solution (HBSS) supplemented with 1,000 U/mL of penicillin, 1,000 μg/mL of streptomycin, 0.25 μg/mL amphotericin B, and 0.04% heat-inactivated FBS. Enzymatic digestion medium was formulated with RPMI 1640 supplemented with 3.33 mg/mL Collagenase (Sigma, #C9407) and 0.5 kU/mL Deoxyribonuclease type IV (Sigma, #D5025).

### Cell lines and constructs

Feeder cells consisted of a modified K562 cell line engineered to express human CD80, CD83, CD86, and CD137L (a gift from the Riley lab at the University of Pennsylvania). Patient-derived cell lines were generated by expanding adherent cells from tumor digests and used for reactivity assays at early passage (<5). B2M knockout (KO) lines were generated by CRISPR-Cas9 using a canine B2M-targeting guide RNA (5’ GAGUACACUUGAAUCUUUGG with Synthego-modified EZ scaffold). MHC class I^-^ populations were identified via flow cytometry and sorted using a BD FACSJazz cell sorter.

### Tumor processing and cell preparation

Tumor tissue samples were obtained from client-owned dogs undergoing clinically indicated surgical procedures either at external institutions (and shipped to our laboratory) or at the Ryan Veterinary Hospital, where samples were provided directly following resection. No animals were anesthetized or euthanized specifically for the purposes of this study.

Patient tumors were collected fresh and transported in tumor transport medium or shipped overnight in enzymatic digest medium. Fresh samples were aseptically minced and incubated overnight in enzymatic digest buffer at room temperature (RT) with gentle rocking. Alternatively, diced tumor fragments (fresh or shipped) were placed in gentleMACS C tubes and dissociated for one hour at 37 °C using a gentleMACS Octo-Dissociator (Miltenyi). Digests were filtered through a 100 μm nylon mesh, washed with Dulbecco’s Phosphate-Buffered Saline (DPBS), and subjected to ACK Lysis Buffer for red blood cell removal. Viable cell counts were performed using a Cellometer and acridine orange/propidium iodide (AOPI) staining. Cells were either used immediately for TIL expansion or cryopreserved in heat-inactivated FBS containing 10% DMSO using a controlled-rate freezing device (Mr. Frosty, Thermo Fisher Scientific).

### TIL expansion and rapid expansion protocol

On day 1, tumor digest was seeded in 24-well plates at 0.5×10^6^ viable cells/mL in TCM supplemented with 3000 IU/mL recombinant human IL-2 (rhIL-2; Biolegend, #589108). TILs were activated with in-house–developed anti-dog CD3/CD28 beads (described previously) at a 1:1 bead-to-cell ratio (74). On day 3, TILs were resuspended at 1×10^6^ cells/mL and transferred to 96-well U-bottom plates. On day 6, beads were removed via magnetic separation, and TILs were re-plated at 1×10^6^ cells/mL in TCM + 3000 IU/mL rhIL-2. Half-media changes were performed every 2–3 days until day 13. For the REP, 1×10^5^ TILs per well were co-cultured with irradiated feeder cells (K562 expressing human CD80, CD83, CD86, and 4-1BBL, irradiated with a gamma irradiator at 10,000 cGy) at a 50:1 feeder-to-TIL ratio in G-Rex24 plates. Cultures were maintained in 7 mL TCM supplemented with 0.5 µg/mL mouse anti-dog CD3 (clone CA17.2A12, BioRad, #MCA1774GA), 3000 IU/mL rhIL-2, and 10 ng/mL recombinant human IL-21 (PeproTech, #200-15). Half-media changes with cytokines were performed on day 19 or 20. TILs were cryopreserved between days 23–24 in heat-inactivated FBS with 10% DMSO. All cultures were incubated at 38.8 °C in a 5% CO_2_ humidified incubator.

### TIL autoreactivity assays

Post-REP TILs were used directly out of culture on day 23–24 or thawed and cultured overnight at 2–4×10^6^ cells/mL in TCM supplemented with 3000 IU/mL rhIL-2 to allow recovery from cryopreservation. The next day, TILs were washed twice in RPMI to wash out cytokines. Autologous tumor digests or early-passage cell lines were thawed, washed, and plated at 1×10^5^ cells per well in 100 µL RPMI CM. TILs were added at 1×10^5^ cells per well (1:1 effector-to-target ratio) in a final 200 µL volume. Conditions included TILs alone, tumor cells alone, and TIL plus tumor co-cultures, all in technical triplicates. Stimulation with cell stimulation cocktail (eBioscience, #00-4970-03) served as a positive control. After 24 h at 38.8 °C in 5% CO_2_, supernatants were collected and analyzed for IFN-γ secretion by ELISA (R&D Systems, #CAIF00). Results are reported as mean ± SD of triplicates.

### Flow cytometry analysis of canine TILs and tumor cells

TILs, tumor digests, and tumor cell lines were washed with FACS buffer (PBS + 2% heat-inactivated FBS). Cells were blocked with 10 µg Dog Gamma Globulin (Jackson ImmunoResearch, #004-000-002) per 1×10^6^ cells for 10 min at RT. For surface staining, cells were incubated with either directly conjugated primary antibodies or unconjugated primary antibodies followed by species-specific fluorophore-conjugated secondary antibodies for 30 minutes at 4 °C in the dark. Intracellular staining was performed after fixation and permeabilization with the Foxp3/Transcription Factor Staining Kit (eBioscience, #00-5523-00) following manufacturer’s instructions, and samples were stained with intracellular antibodies for 45 min at RT. Cells were washed three times in FACS buffer before acquisition on a BD LSRFortessa and data analysis using TreeStar FlowJo v10.8. Gating was performed using fluorescence-minus-one or isotype control samples. Antibody information is provided in **Table 2**.

**Table 2:** Flow antibody reagent list.

Target	Clone	Fluorophore	Catalogue #	Company
Rat anti-dog CD5	YKIX322.3	APC-eF780	47-5050-42	Invitrogen
Rat anti-dog CD5	YKIX322.3	PE	12-5050-42	Invitrogen
Rat anti-dog CD4	YKIX302.9	PB	MCA1038PB	BioRad
Rat anti-dog CD8α	YCATE55.9	FITC	MCA1039F	BioRad
Mouse anti-dog CD25	P4A10	FITC	MCA5916F	BioRad
Mouse anti-dog CD25	P4A10	eFlour 660	50-0250-42	eBioscience
Mouse anti-dog CD3	CA17.2A12	Unconjugated	MCA1774GA	BioRad
Mouse anti-dog CD94	8H10	AF647	MCA6400A647	BioRad
Mouse anti-dog MHC Class I	H58A	Unconjugated	NBP2-61002	Novus Biologicals
Mouse anti-dog CD28	5B8	APC	17-0282-42	eBioscience
Mouse anti-human CD62L	FMC46	PE	MCA1076PE	BioRad
Mouse anti-human CD62L	FMC46	StarBright Blue 675	MCA1076SBB675	BioRad
Armenian hamster anti-mouse/rat/human CD27	LG.7F9	BV786	417-0271-82	Invitrogen
Rat anti-dog MHC Class II	YKIX334.2	APC	17-5909-42	eBioscience
Mouse anti-canine Melan-A	SPM555	Unconjugated	NBP2-34797	Novus Biologicals
Armenian hamster anti-mouse/human Helios	22F6	PerCp/Cyanine5.5	137230	BioLegend
Rat anti-dog FOXP3	FJK-16s	APC	17-5773-82	eBioscience
Fixable live/dead	n/a	Aqua	L34957	Invitrogen
Fixable live/dead	n/a	Violet	L34964	Invitrogen
Fixable live/dead	n/a	Yellow	L34967	Invitrogen
Anti-mouse IgG	Polyclonal	PE	405307	Biolegend
Anti-mouse IgG	Polyclonal	FITC	405305	Biolegend

### Statistics

All statistical analyses were performed in GraphPad Prism 9. Data were evaluated for normality using the Shapiro–Wilk test prior to selecting parametric or nonparametric statistical tests. Proportional data were arcsine square-root transformed prior to analysis to stabilize variance. Paired comparisons between conditions were performed using a paired t-test or Wilcoxon signed-rank test, dependent on normality testing and presence of outliers. Group differences were analyzed using an ordinary one-way ANOVA followed by Tukey’s multiple comparisons test. Reported p-values reflect Tukey-adjusted values. Where multiple t-tests were performed, p-values were adjusted for multiple comparisons using the Benjamini–Krieger–Yekutieli (BKY) two-stage false discovery rate (FDR) method. Significance annotations in figures correspond to the resulting FDR-adjusted p-values. For correlation analysis, tumor reactivity was expressed as the log_2_ fold change in IFN-γ secretion measured during co-culture with autologous tumor relative to baseline TIL culture. To mitigate instability from low baseline IFN-γ values, a small pseudocount equal to the limit of detection of the ELISA was added to all measurements prior to log_2_ transformation. Associations between tumor reactivity and TIL expansion were evaluated using Spearman’s rank correlation. Asterisks indicate significance levels (* p < 0.05; ** p < 0.01; *** p < 0.001; **** p < 0.0001; ns, not significant).

## Data Availability

The raw data supporting the conclusions of this article will be made available by the authors, without undue reservation.
