# Supplementary material for: Generation of functional canine TIL products for solid tumors
Source: Front Immunol. 2026 May 13;17:1810955. doi: 10.3389/fimmu.2026.1810955 (PMC13212238; doi:10.3389/fimmu.2026.1810955)
Supplement: Supplementary file 1 [file DataSheet1.pdf]

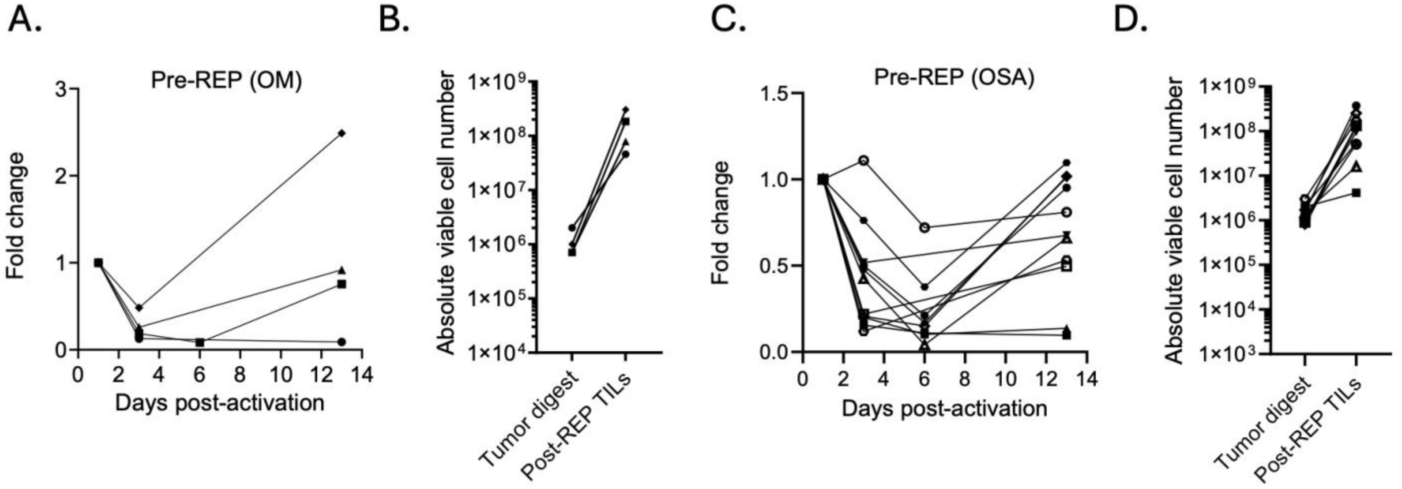

Supplemental Figure 1. TIL expansion dynamics across pre-REP and REP phases in melanoma and osteosarcoma cohorts.

(A) Pre-REP expansion of melanoma-derived TILs, shown as fold change relative to initial plating conditions. This panel corresponds to Figure 1B, which displays the same samples as absolute viable cell counts. **(B)** Total viable cell expansion from the melanoma tumor digest to the post-REP product. This panel corresponds to Figure 1F, which presents $CD5^+$ T cell expansion over the same time course. **(C)** Pre-REP expansion of osteosarcoma-derived TILs, shown as fold change relative to initial plating conditions. This panel corresponds to Figure 5B, which displays the same samples as absolute viable cell counts. **(D)** Total viable cell expansion from osteosarcoma tumor digest to post-REP product. This panel corresponds to Figure 5F, which presents $CD5^+$ T cell expansion over the same time course.

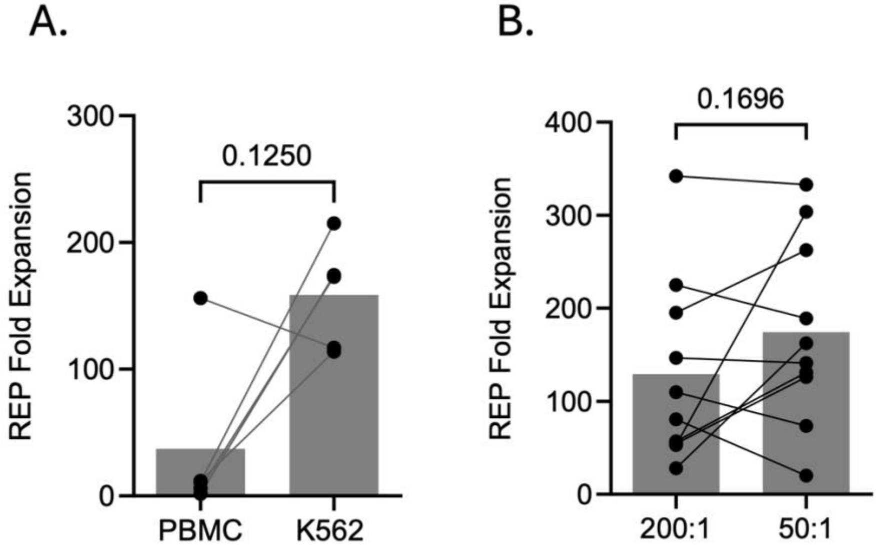

Supplemental Figure 2. TILs reliably expand in the presence of K562 feeder cells.
Growth kinetics of OM and OSA TILs during REP. TILs were co-cultured with irradiated feeder cells, 3000 IU/mL IL-2, and anti-dog CD3. Data show the total fold change in TILs over the course of the REP. **(A)** TILs from two OSA donors were co-cultured with canine PBMCs or K562 feeder cells (n = 5 paired cultures; three at 50:1 and two at 200:1 feeder-to-TIL ratios). **(B)** TILs from 10 donors (4 OM, 6 OSA) were co-cultured at 200:1 and 50:1 feeder-to-TIL ratios using K562 feeder cells. Statistical significance was determined by **(A)** Wilcoxon signed-rank and **(B)** paired two-tailed t-tests.

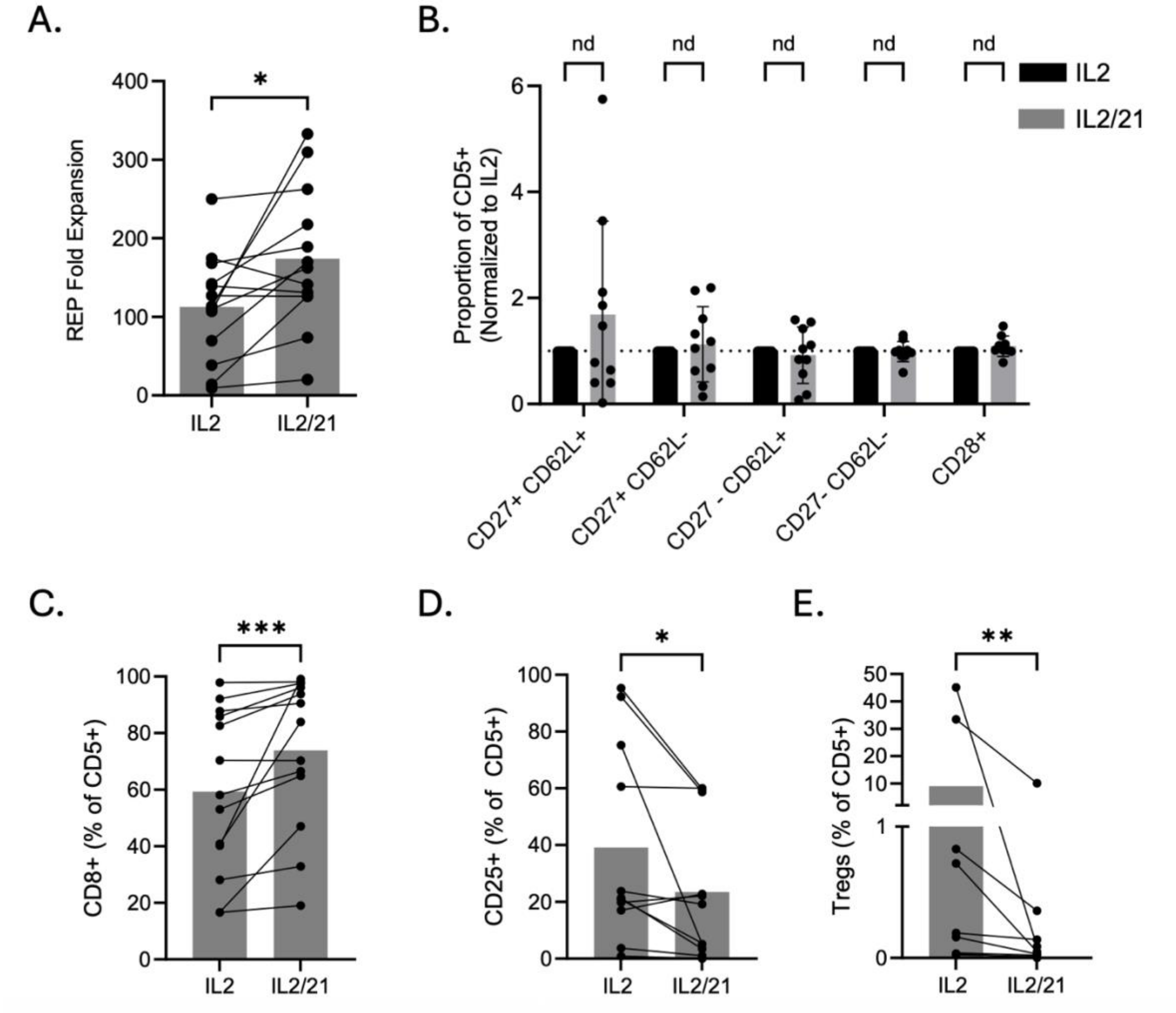

Supplemental Figure 3. IL-21 supplementation enhances CD8$^+$ TIL expansion but does not alter memory phenotype.

TILs were co-cultured with irradiated feeder cells, 3000 IU/mL IL-2, and anti-dog CD3 with and without 10 ng/mL IL-21 supplementation. Analysis was performed on day 23 or day 24 TILs (post-REP). **(A)** Data show the fold change in OM and OSA TILs over the course of the REP (n = 13). **(B)** Proportion of CD5$^+$ post-REP TILs with memory-associated phenotypes defined by CD27 and CD62L expression (n = 10) and costimulatory receptor CD28 expression (n = 8). Proportions were normalized to IL2 to account for patient variability. **(C-E)** Proportion of **(C)** CD8$^+$ subsets (n = 13), **(D)** CD25$^+$ subsets (n = 11), and **(E)** CD4$^+$CD25$^+$Helios$^+$FOXP3$^+$ Treg subsets (n = 8) within CD5$^+$ post-REP TILs. Statistical significance was determined by **(A,D)** paired two-tailed t-tests, (**B)** multiple paired t-tests with BKY FDR correction, and **(C,E)** Wilcoxon signed-rank tests. Each point represents an individual dog; proportions are represented as the mean ± SD. Asterisks indicate significance levels (* $p < 0.05$; ** $p < 0.01$; *** $p < 0.001$). nd indicates no statistical difference after FDR correction.

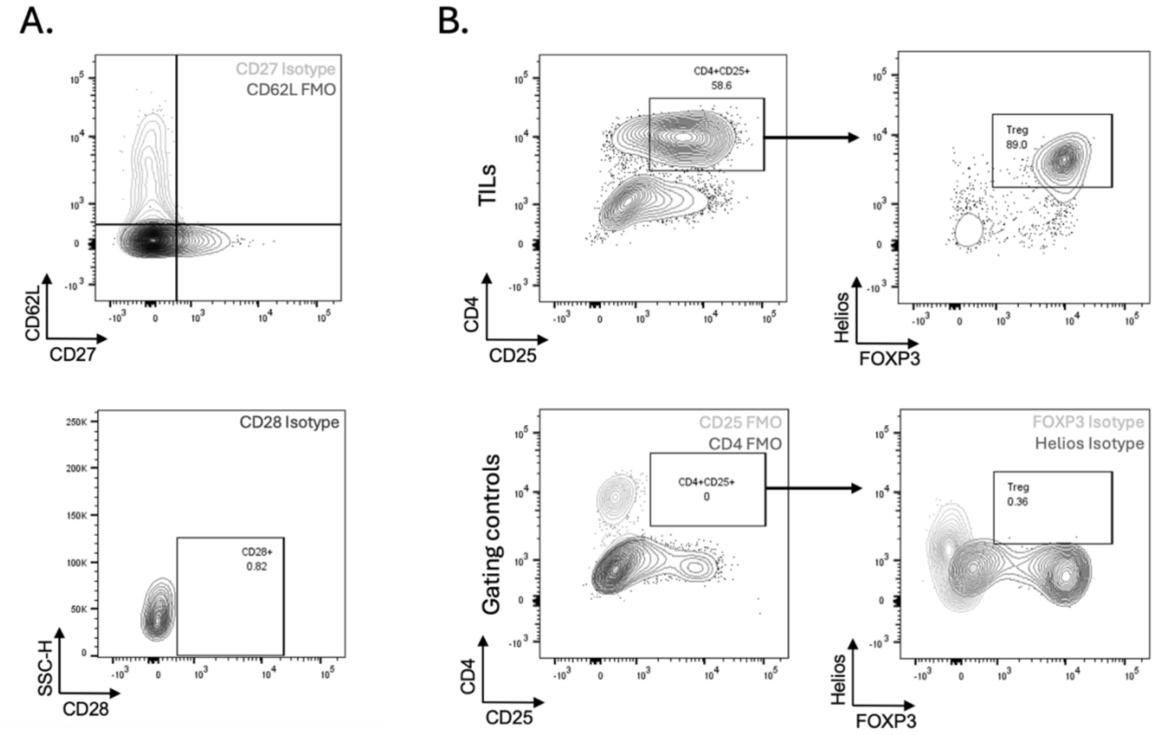

Supplemental Figure 4. Gating strategy and validation of flow cytometry controls for T cell subset analysis.
(A) Representative gating strategy for identification of T cell memory subsets based on CD62L, CD27, and CD28 expression within CD5$^+$ T cells. Isotype and fluorescence minus one (FMO) controls were used to establish gating boundaries for each marker. **(B)** Representative staining and gating of regulatory T cells (Tregs) within a TIL product generated in the absence of IL-21, which exhibited a higher frequency of Treg-like cells. Tregs were identified based on CD4, CD25, FOXP3, and HELIOS expression, and isotype controls and FMOs were used to establish gating boundaries, as displayed in the lower panel.

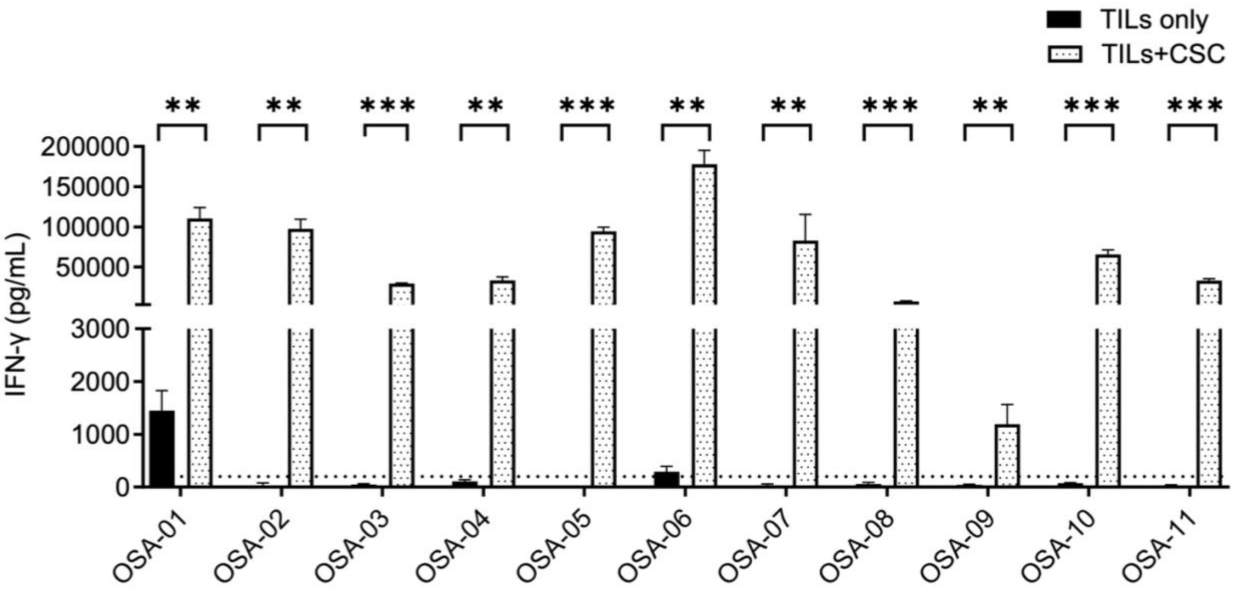

Supplemental Figure 5. All canine OSA TIL products are functional.

1×10^5 post-REP TILs were seeded per well in 96-well plates and treated with (stippled bars) or without (black bars) cell stimulation cocktail (CSC) for 24 hours. IFN-γ secretion was measured by ELISA ($n = 11$). Experiments were performed in triplicate wells per condition. Multiple t-tests were performed, and p-values were adjusted for multiple comparisons using the BKY two-stage FDR method. Significance annotations correspond to the resulting FDR-adjusted p-values (q). Results are reported as mean ± SD of triplicates. Asterisks indicate significance levels (** $p < 0.01$, *** $p < 0.001$).

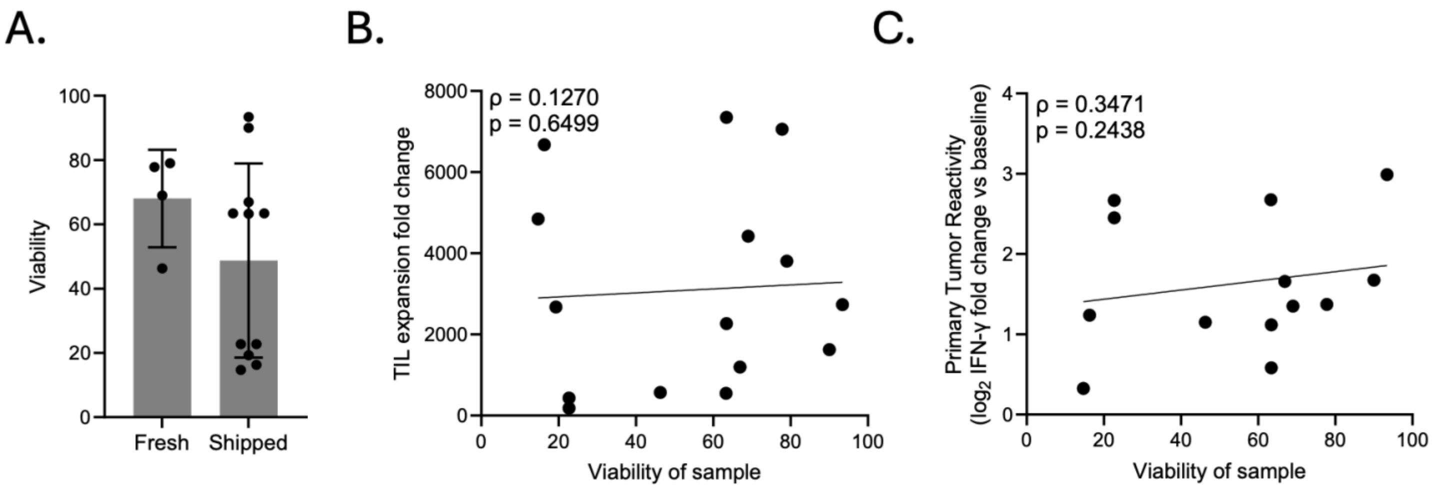

Supplemental Figure 6. Impact of tumor digest viability on TIL expansion and functional activity
(A) Viability of tumor digests following processing, comparing fresh (n = 4) versus overnight-shipped (n = 11) samples. **(B)** Correlation between tumor digest viability and $CD5^+$ TIL expansion fold change across the entire culture duration (n = 15). **(C)** Correlation between tumor digest viability and tumor reactivity of expanded TILs. Tumor reactivity was quantified as the $\log_2$ fold change in IFN-γ secretion by expanded TILs following co-culture with autologous primary tumor digests (n = 13). Melanoma and osteosarcoma samples are combined for analysis. Spearman's rank correlation coefficient (ρ) and associated p-value are shown.
